# The Joint Association of Diet Quality and Sleep Regularity with Incident Cardiovascular Disease in the Multi-Ethnic Study of Atherosclerosis

**DOI:** 10.3390/nu17111750

**Published:** 2025-05-22

**Authors:** Kaitlin S. Potts, Claire Veldkamp, Alexis C. Wood, Erin D. Michos, Raymond Noordam, Tianyi Huang, Susan Redline, Heming Wang

**Affiliations:** 1 Division of Sleep and Circadian Disorders, Department of Medicine, Brigham and Women’s Hospital, Boston, MA 02115, USA; c.c.veldkamp@student.vu.nl (C.V.); sredline@bwh.harvard.edu (S.R.); 2 USDA/ARS Children’s Nutrition Research Center, Baylor College of Medicine, Houston, TX 77030, USA; alexis.wood@bcm.edu; 3 Division of Cardiology, Johns Hopkins University School of Medicine, Baltimore, MD 21205, USA; edonnell@jhmi.edu; 4 Department of Internal Medicine, Section of Gerontology and Geriatrics, Leiden University Medical Center, 2300 RC Leiden, The Netherlands; r.noordam@lumc.nl; 5 Health Campus The Hague, Department of Public Health and Primary Care, Leiden University Medical Center, 2511 DP The Hague, The Netherlands; 6 Laboratory of Epidemiology and Population Sciences, Intramural Research Program, National Institute on Aging, Baltimore, MD 21224, USA; tianyi.huang@nih.gov

**Keywords:** sleep health, sleep regularity, diet quality, dietary patterns, cardiovascular disease

## Abstract

Background/Objectives: Diet quality and sleep regularity both influence cardiovascular disease (CVD) risk and may influence each other, but there is scarce evidence for their joint or interacting associations in relation to CVD. We assessed these associations in the Multi-Ethnic Study of Atherosclerosis (MESA). Methods: Participants free of CVD with valid diet and sleep measures in 2010–2013 were included and followed through 2020 for detection of incident CVD (188 events detected over 8.8 years among 1782 participants; 55% women). Sleep timing and duration regularity were assessed via the intra-individual SD of sleep onset time and duration across 5- to 7-day actigraphy. The Alternate Healthy Eating Index-2010 assessed diet quality. Sleep regularity and diet quality were dichotomized and cross-tabulated to estimate joint associations with CVD and to evaluate interaction via Cox proportional hazard models adjusting for potential confounders. Results: Participants with low diet quality and irregular sleep had higher CVD risk compared to those with high diet quality and regular sleep (adjusted HR [95% CI]: low diet quality + irregular sleep timing: 1.56 [1.03, 2.37]; low diet quality + irregular sleep duration: 1.70 [1.09, 2.67]). The joint associations were stronger than those for only one adverse behavior and similar to those for their combination. There was no evidence for additive or multiplicative interactions. Conclusions: Having irregular sleep and low diet quality confers the highest CVD risk compared to having neither or only one of these behaviors. These results underscore the importance of interventions targeting these unhealthy lifestyle behaviors, especially when they co-occur.

## 1. Introduction

Healthy sleep and dietary behaviors are recognized as essential components of a lifestyle that reduce risk of cardiovascular disease (CVD), the primary cause of death in the United States and globally [1]. Regularly eating a healthy diet is a well-established protective behavior for cardiovascular and other chronic diseases [2]. Insufficient and poor quality sleep is also associated with increased CVD risk [3]. Other research has highlighted the impact of circadian disruption and misalignment on cardiometabolic health outcomes, as evidenced by studies in shift workers, those with social jetlag or late chronotypes, and experimental settings on forced sleep deprivation [4,5,6,7]. Irregular sleep timing or duration may be a marker of circadian disruption or misalignment in the broader population. These findings highlight the importance of sleep regularity, beyond duration and quality, for cardiometabolic health, as concluded by a recent consensus panel of the National Sleep Foundation [8]. Studies have shown that irregular sleep, measured by variability in sleep duration or timing, is associated with CVD risk factors, incident CVD, and mortality [9,10,11,12,13].

There is also emerging evidence that sleep and diet may bidirectionally influence each other, highlighting the potential joint influence of multiple lifestyle behaviors on CVD risk [14,15]. Experimental sleep restriction associates with poor next-day dietary choices (e.g., increased preference for sweets and higher total energy foods), and both short and poor quality sleep (including irregular sleep) associate with low diet quality in observational cohorts [16,17,18,19,20,21,22]. In the other direction, certain foods, dietary patterns, and macronutrient profiles may influence sleep [23,24,25,26,27]. The potential that these behaviors influence each other suggests that these behaviors may frequently co-occur. In addition, poor dietary and sleep habits share common social and environmental determinants, furthering the potential for these adverse health behaviors to cluster together. Since diet quality and sleep regularity both influence cardiovascular health and often co-occur, it is important to quantify their joint effects on CVD risk—especially given the potential population-level impact of this behavioral pattern. Yet, very few studies have attempted to estimate these joint effects. One study conducted in the National Health and Nutrition Examination Survey found an increased risk of all-cause and cardiovascular mortality among those with sleep disorders and low diet quality [28]. However, the findings were limited to self-reported sleep data and did not assess associations with non-fatal CVD, nor were potential interactive effects fully investigated. A clearer understanding of how these behaviors jointly influence CVD risk, and if there are synergistic or antagonistic interactions, could inform intervention targeting and resource allocation towards the most at-risk patients. This knowledge may also support the development of multi-component interventions and lead to more effective treatment strategies for the prevention of CVD.

The objective of this study was to investigate the joint impact of sleep regularity (based on timing and duration) and diet quality on CVD risk and to test for potential interaction effects between these behaviors. We hypothesized that individuals with poor diet quality and irregular sleep have the highest risk of incident CVD compared to those with healthier diets and more regular sleep, or only one risk behavior.

## 2. Materials and Methods

### 2.1. Study Population

**Figure 1:**
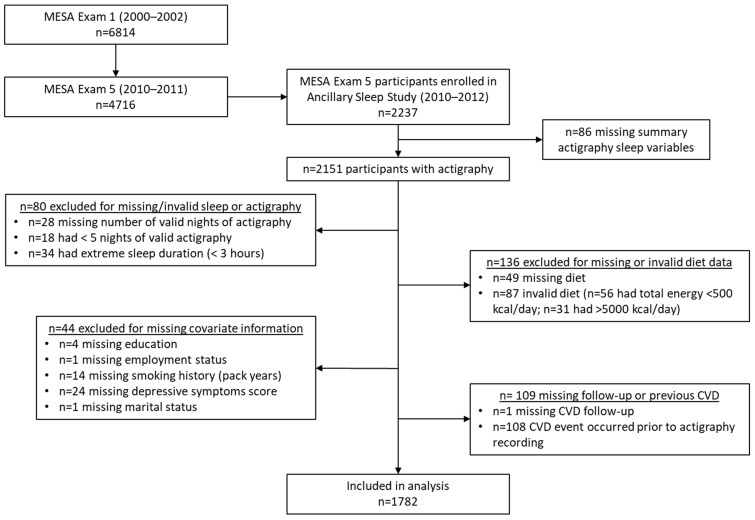
Participant flowchart.

The Multi-Ethnic Study of Atherosclerosis (MESA) is a longitudinal community-based cohort investigating cardiovascular risk. The first visit (2000–2002) enrolled 6814 participants aged 45–84 years with no history of CVD from six U.S. sites (Baltimore, MD; Chicago, IL; Forsyth County, NC; Los Angeles, CA; New York, NY; St. Paul, MN). The baseline racial and ethnic distribution was 38% White, 28% Black, 22% Hispanic/Latino, and 12% Chinese individuals [29]. There have been 7 exams, and participants are actively followed for cardiovascular events. At Exam 5 (2010–2012), 4716 participants completed a dietary assessment, and those not using oral devices, nocturnal oxygen, or positive airway pressure were invited to join the MESA Sleep Study. Among 3789 eligible participants, 2237 enrolled, completing 7-day actigraphy and standardized sleep questionnaires [30]. This analysis excluded those with missing summary sleep measures (n = 86), <5 (n = 18) or missing (n = 28) number of valid nights, <3 h average sleep duration (n = 34), missing or invalid diet (n = 136), or missing covariates (n = 54) (Figure 1). Additionally,108 were excluded for having a CVD event prior to the sleep exam, and 1 person was lost to follow-up, leaving 1782 participants for this analysis. All participants provided written informed consent at each visit, and the methods were approved by institutional review boards at each site.

### 2.2. Sleep Assessment

Participants wore the Actiwatch Spectrum (Philips Respironics, Murrysville, PA, USA) on their non-dominant wrist and recorded bed/wake times in sleep diaries for seven days. Actigraphy data were processed using Actiware-Sleep (v5.59), which classified 30 s epochs as sleep or wake via a validated algorithm [31]. The start and end of the main sleep period were marked by a certified technician at the Brigham and Women’s Hospital Sleep Reading Center using a standardized method incorporating the event marker, sleep diary, light intensity, and activity counts [30]. Sleep duration was calculated as the total time spent asleep (sum of sleep epochs) within the main sleep period. Sleep regularity was assessed using the intra-individual SD of sleep onset time (sleep timing regularity, SD sleep onset) and sleep duration (sleep duration regularity, SD sleep duration) across the recording period (see Supplementary Figure S1 for correlations). Based on prior research and sample size considerations, sleep timing regularity was classified as regular (<60 min SD sleep onset) or irregular (≥60 min SD sleep onset), and sleep duration regularity was classified as regular (<90 min SD sleep duration) or irregular (≥90 min SD sleep duration) [9]. Additional sleep characteristics were captured via validated questionnaires, including excessive daytime sleepiness [32], insomnia symptoms [33], and chronotype [34,35]. See Supplementary Figures S1–S4 for correlations and distributions of sleep phenotypes.

### 2.3. Diet Assessment

Diet was measured at Exam 5 with a food frequency questionnaire (FFQ), where participants reported commonly consumed foods, frequency, and portion size to estimate usual dietary intake [36]. The MESA FFQ was adapted for the multi-ethnic sample by including culturally relevant foods and was validated against eight 24 h recalls over the same one-year period, showing correlations similar to other FFQs [36,37,38]. The FFQ included 128 food and drink items using 9 frequency options (rarely/never to ≥2 times/day) and 3 serving sizes [27]. Nutrient and food group (e.g., fruits, vegetables, and dairy) intakes were estimated with the Nutrient Data System for Research. From these, we derived the Alternate Healthy Eating Index 2010 (AHEI) as a measure of overall diet quality. The AHEI is inversely associated with chronic disease risk, including cardiovascular mortality [39]. It includes eleven components (vegetables, fruits, whole grains, sugar-sweetened beverages and fruit juice, nuts/legumes, red/processed meat, trans fats, long-chain n-3 fats, polyunsaturated fats, sodium, and alcohol), each scored 0–10 based on pre-defined optimal intakes for health. The summed total score ranges from 0 to 110 (higher score indicates a healthier diet). See Supplementary Table S1 for the scoring details. Diet quality was dichotomized to high/low quality based on the median AHEI score (see distribution in Supplementary Figure S5).

### 2.4. Ascertainment of Cardiovascular Endpoints

Participants were contacted every 9–12 months to report hospitalizations, cardiovascular diagnoses, procedures, and deaths [29]. Additional events were ascertained through cohort visits, patient call-ins, medical records, or obituaries. Eligible self-reported events underwent medical record abstraction by cardiovascular physician epidemiologists or neurologists [40]. Two physicians working independently reviewed all endpoints, and any disagreements underwent full committee review. Nonfatal events included coronary heart failure, angina, myocardial infarction, resuscitated cardiac arrest, peripheral artery disease, stroke, and transient ischemic attack. Fatal events included fatal coronary heart disease, fatal stroke, and other fatal CVD. All deaths were identified, and deaths possibly related to CVD underwent committee review. For increased statistical power, we included all fatal and nonfatal cardiovascular events as the study outcome.

### 2.5. Assessment of Covariates

Participants self-reported their birth date, sex, ethnicity, and education level (<high school, high school degree, come college, and bachelor’s degree or higher) at Exam 1. We used Exam 5 data for characterizing current employment status (employed/not employed), marital status (married/not married), smoking history (pack-years), physical activity (MET hours/week) [41], and depressive symptoms [42]. Season of actigraphy recording was classified as winter, spring, summer, or fall. Work schedule, reported on the sleep questionnaire, classified night, split, irregular/on-call, and rotating shifts as shift workers. Cardiometabolic risk factors at Exam 5 included BMI (kg/m^2^), diabetes status (normal, impaired fasting glucose, untreated diabetes, and treated diabetes) [43], HbA1c, non-HDL cholesterol (mg/dL), and systolic blood pressure (SBP) [44].

### 2.6. Statistics and Reproducibility

We used multivariable Cox proportional hazards regression models to estimate adjusted hazard ratios (HR) and 95% confidence intervals (CI) for incident CVD by sleep regularity, diet quality, and their joint effects. Time was calculated as the number of days from the start of actigraphy recording to CVD event, death, loss, or end of follow-up (31 December 2020), whichever came first. We adjusted for a pre-determined set of covariates in a series of nested models. Model 1 included age, sex, race, and ethnicity. Model 2 additionally included total energy intake and height, education, employment status, marital status, study site, and season of actigraphy. Model 3 further included physical activity, smoking history, and depressive symptoms. In the sensitivity models, we adjusted for shiftwork (a potential cause of irregular sleep), other CMD risk factors (potential mediators), and other sleep characteristics (to interrogate independence of detected associations from other sleep factors).

We followed the method of VanderWeele and Knol, using a common reference group to estimate individual and joint HRs via a 2 × 2 matrix of binary variables, facilitating comparative interpretation and assessment of additive and multiplicative interaction (see Supplementary Table S2) [45]. Additive interaction was estimated by the relative excess risk due to interaction (RERI), calculated as the difference between the sum of the individual HRs and the joint HR (if different from 0, there is evidence of additive interaction) [46,47,48]. Multiplicative interaction was evaluated by determining if the ratio of the product of the individual HRs and the joint HR differed from 1. We used R package interactionR to estimate these parameters [49] and the variance recovery method to obtain the 95% confidence intervals [50] (p. 201). Multiplicative interaction for continuous exposure was assessed by including product terms for AHEI and SD sleep duration/timing in the models. We additionally estimated HRs for diet quality and sleep regularity within strata of each other, as recommended by VanderWeele and Knol [45]. In the exploratory analysis, we visualized smoothed joint associations using the continuous measures of diet quality and sleep regularity, using R package mgcv to estimate the smoothed effects with generalized additive models (gam function, cox.ph family) [51].

In accordance with recommendations from the American Statistical Association, we did not interpret the results in relation to *p*-values or binary statistical significance testing [52]. Rather, we focused on the magnitude, direction, precision, and consistency of estimates to learn from the data.

## 3. Results

### 3.1. Description of Sample

Among the 1782 participants included in this analysis, 55% were female and the mean age was 68 years (Table 1). There were 188 CVD events over 14,323 person years of follow-up (median [IQR]: 8.8 [1.29] years of follow-up). The ethnic diversity was similar to the entire MESA cohort, with 39% White, 12% Chinese, 27% Black, and 22% Hispanic/Latino. About 41% had a bachelor’s degree or higher, 61% were currently married, and 25% were employed. The average sleep duration was 6.6 h (SD 1.2), and the intra-individual SD of nightly sleep duration was 75 min (SD 37.5). Overall, 30.6% were classified as having irregular sleep duration. The intra-individual SD of sleep onset was 80 min (SD 93), and 46% were classified as having irregular sleep timing. The mean AHEI score was 59.3 (SD 10.8), and 48% were classified as having low diet quality.

After cross-tabulation to assess the joint distribution of diet quality and sleep timing regularity, 30% had regular sleep timing and high diet quality (reference group), and 23% had irregular sleep timing and low diet quality (Table 2). For sleep duration regularity, the distribution included 36% with high diet quality and regular sleep duration (reference group) and 15% with low diet quality and irregular sleep duration.

### 3.2. Individual Associations

CVD-free survival was higher among those with higher diet quality, and among those with more regular sleep, although the 95% CIs of the survival curves overlapped (Supplementary Figures S6–S9). In the adjusted Cox models, each one-SD increase in AHEI was associated with a 12% lower hazard of CVD (model 3 HR [95% CI]: 0.88 [0.75, 1.04]), and being in the low diet quality group, compared to high, had a 35% higher hazard of CVD (model 3 HR [95% CI]: 1.35 [0.99, 1.84]) (Table 3). Similar trends were observed when AHEI quintiles were used, where those in quintile 1 of AHEI (lowest diet quality) had higher risk compared to those in quintile 5 (highest diet quality) (e.g., model 3 HR [95% CI] for quintile 1 vs. 5: 1.35 [0.86, 2.12]). Each one-hour increase in SD sleep onset was associated with a 10% higher risk of CVD (model 3 HR [95% CI]: 1.10 [1.02, 1.19]), while each one-hour increase in SD sleep duration was associated with a 24% higher hazard of CVD (model 3 HR [95% CI]: 1.24 [0.98, 1.71]). These trends were similar when dichotomized or categorized versions of the sleep regularity variables were used (model 3 HR [95% CI]: for irregular sleep timing, SD ≥ 60 min vs. less: 1.21 [0.91, 1.63]; for irregular sleep duration, SD ≥ 90 min vs. less: 1.25 [0.92, 1.71]). While the 95% CIs indicate imprecision, these estimates remained very consistent across models, including with further adjustments in sensitivity analyses (Supplementary Table S3).

### 3.3. Joint and Individual Associations in Comparison to a Common Reference Group

**Figure 2:**
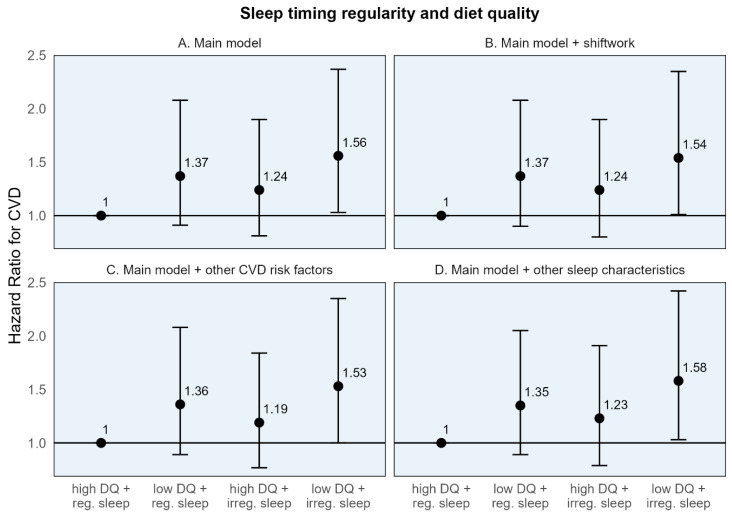
Adjusted hazard ratios and 95% confidence intervals for incident cardiovascular disease for sleep timing regularity, diet quality, and their joint effect compared to a common reference group. CVD: Cardiovascular disease. High diet quality (high DQ): AHEI-2010 ≥ median value; low diet quality (low DQ): AHEI-2010 < median value. Regular sleep timing (reg. sleep): SD sleep onset time < 60 min; irregular sleep timing (irreg. sleep): SD sleep onset time ≥ 60 min. (**A**) Main model adjusted for age, sex, race/ethnicity + total energy intake (kcal/d; diet models only), height (cm; diet models only), education (<high school, high school degree, some college, and bachelor’s degree or higher), employment status (employed/unemployed), marital status (currently married/single, divorced, and widowed), study site, season of actigraphy recording (sleep models only; winter, spring, summer, and fall) + physical activity (self-report, MET-hours per week), smoking history (pack-years), depressive symptoms (CES-D ≥ 16). (**B**) Sensitivity model additionally adjusting for shiftwork. (**C**) Sensitivity model additionally adjusting for CVD risk factors: BMI, HbA1c, non-HDL cholesterol, and systolic blood pressure. (**D**) Sensitivity model additionally adjusting for other sleep characteristics: insomnia symptoms, chronotype, excessive daytime sleepiness, and average sleep duration.

**Figure 3:**
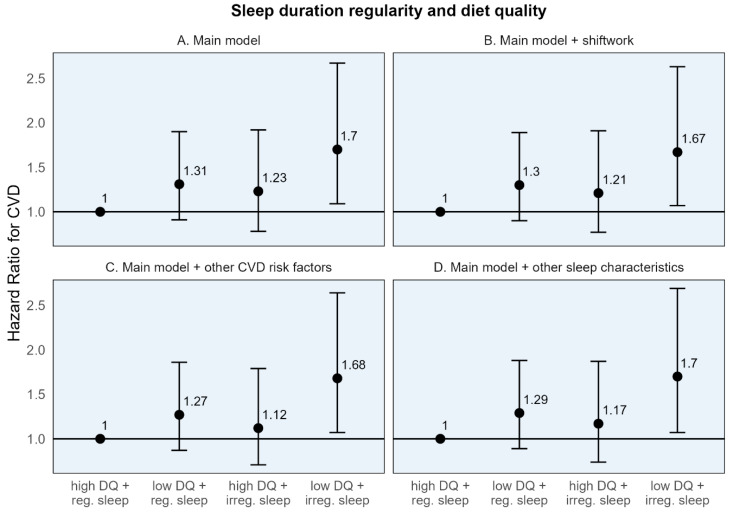
Adjusted hazard ratios and 95% confidence intervals for incident cardiovascular disease for sleep duration regularity, diet quality, and their joint effect compared to a common reference group. CVD: Cardiovascular disease. High diet quality (high DQ): AHEI-2010 ≥ median value; low diet quality (low DQ): AHEI-2010 < median value. Regular sleep timing (reg. sleep): SD sleep onset time < 60 min; irregular sleep timing (irreg. sleep): SD sleep onset time ≥ 60 min. (**A**) Main model adjusted for age, sex, race/ethnicity + total energy intake (kcal/d; diet models only), height (cm; diet models only), education (<high school, high school degree, some college, and bachelor’s degree or higher), employment status (employed/unemployed), marital status (currently married/single, divorced, and widowed), study site, season of actigraphy recording (sleep models only; winter, spring, summer, and fall) + physical activity (self-report, MET-hours per week), smoking history (pack-years), depressive symptoms (CES-D ≥ 16). (**B**) Sensitivity model additionally adjusting for shiftwork. (**C**) Sensitivity model additionally adjusting for CVD risk factors: BMI, HbA1c, non-HDL cholesterol, and systolic blood pressure. (**D**) Sensitivity model additionally adjusting for other sleep characteristics: insomnia symptoms, chronotype, excessive daytime sleepiness, and average sleep duration.

When participants were classified into four groups based on high/low diet quality and regular/irregular sleep, CVD-free survival was highest throughout the follow-up period among the group with high diet quality and regular sleep for both sleep timing and duration regularity (Figures S10 and S11). While those with poor diet quality and irregular sleep had the lowest CVD-free survival through most of the follow-up period. In the adjusted Cox models, the risk for CVD was highest in those with both adverse exposures—low diet quality and irregular sleep—for both sleep timing and duration regularity. Those with low diet quality and irregular sleep timing had a 56% higher risk of CVD (HR: 1.56, 95% CI: 1.03, 2.37), and those with low diet quality and irregular sleep duration had a 70% higher risk of CVD (HR [95% CI]: 1.70 [1.09, 2.67]) compared to the common reference group (Table 4). While somewhat imprecise, these estimates remained stable, with additional adjustment for shiftwork, CMD risk factors, and other sleep characteristics (Figure 2 and Figure 3).

The individual HRs for the dichotomized diet quality and sleep regularity variables, in relation to the common reference group, demonstrated a numerically larger association for poor diet quality compared to irregular sleep. For example, compared to the common reference of high diet quality and regular sleep timing, the hazard of CVD was 37% higher [95% CI: 0.91, 2.08] for those with poor diet quality, compared to 24% higher [95% CI: 0.81, 1.90] for those with irregular sleep timing (Table 4). A similar pattern was seen for sleep duration regularity. However, the 95% CIs are somewhat wide, indicating some imprecision.

These results are similarly reflected in the stratum-specific HRs (the estimated association of one behavior within the stratum of the other) where the association between low (vs. high) diet quality with CVD was numerically larger in both strata of sleep timing regularity (HRs: 1.37 and 1.26 among those with high and low diet quality, respectively) compared to the association of irregular (vs. regular) sleep in either strata of diet quality (HRs: 1.24 and 1.14 among those with regular and irregular sleep timing, respectively) (Table 4). Similar findings were seen for sleep duration regularity.

### 3.4. Interaction

We did not find evidence of additive or multiplicative interaction. The *p*-value for the interaction product term using continuous variables was 0.340 for SD sleep onset time × AHEI and 0.805 for SD sleep duration × AHEI (Table 4). The multiplicative interactions assessed via the ratio of the product of the individual HRs to the joint HR were close to 1, the null value (0.92 [95% CI: 0.51, 1.64] and 1.06 [95% CI: 0.58, 1.96] for sleep timing and duration regularity, respectively). The RERIs, comparing the difference between the joint HRs and the sum of the individual HRs to assess additive interaction, were close to 0, the null value (−0.05 [95% CI: −1.00, 0.66] and 0.17 [95% CI: −0.78, 1.04] for sleep timing and duration regularity, respectively). The lack of evidence for interaction reflects that the joint HRs did not differ from what would be expected based on the individual HRs, all estimated in relation to a common reference group.

In the exploratory analysis, the smoothed main and interaction terms using continuous diet quality and sleep regularity variables were plotted to visualize the risk pattern using continuous variables (Supplementary Figures S12 and S13). These plots revealed similar results, where the highest risk was found among those with low diet quality and irregular sleep, and suggested that the interaction terms had minimal impact.

## 4. Discussion

We investigated the combined association of sleep regularity and diet quality on total CVD incidence in an older multi-ethnic cohort. Participants with the combination of irregular sleep and low diet quality had higher CVD risk compared to those with regular sleep and high diet quality and compared to having only one of these adverse behaviors. Specifically, those with low diet quality and irregular sleep timing had a 56% higher hazard of CVD, and those with low diet quality and irregular sleep duration had a 70% higher hazard of CVD, compared to individuals with high diet quality and regular sleep. In contrast, the hazard ratios for individual adverse diet or sleep behaviors did not exceed 1.37, or a 37% higher CVD risk. These results were unchanged after adjusting for traditional CMD risk factors and other sleep characteristics. There was no evidence of interaction on additive or multiplicative scales.

While imprecise, the point estimates for the individual HRs for CVD for diet quality and sleep regularity were in alignment with the results from previous studies. For example, the HR per one SD increase in AHEI was 0.88 (95% CI: 0.75, 1.04) and the HR for being in the highest vs. lowest quintile of AHEI was 0.74 (95% CI: 0.47, 1.16). These estimates are similar to those from a meta-analysis of 21 reports that found that the pooled relative risk for CVD comparing those in the highest vs. lowest AHEI category was 0.77 (95% CI: 0.74, 0.80) [2]. A previous MESA analysis established sleep irregularity as a risk factor for incident CVD. Our results were similar, including finding a larger, but less precise, association between SD sleep duration and CVD compared to SD sleep onset time and CVD [9]. The estimates in this analysis were slightly smaller but more precise compared to the previous study. The differences are likely due to differential exclusion criteria given the additional focus on diet quality and an additional two years of follow-up.

Our results indicated a slightly larger individual association between low diet quality and CVD compared to irregular sleep and CVD when using the same reference group. While interesting, this finding should not be over-interpreted since we cannot directly compare the estimates in terms of the levels used to define low diet quality and irregular sleep, which are intrinsically different. For example, the comparison may have differed if irregular sleep duration was classified as those with >120 min of SD sleep duration or if low diet quality captured individuals only in the lowest quintile of AHEI.

This study does not offer evidence for whether sleep regularity and diet quality influence one another but supports the hypothesis that those who are doubly burdened by irregular sleep and poor diet quality face the highest risk of CVD compared to exposure to neither or just one of these behaviors. While not the aim of this study, the hypothesis that these behaviors reinforce one another is relevant for interpretating these results. Prior studies support the associations between diet quality and poor sleep quality, which may include irregular sleep [17,18,19,20,21,22,23,27,53,54,55]. In addition, irregular sleep may influence diet quality indirectly through altered meal timing, which has been associated with lower diet quality and increased cardiometabolic risk markers [56,57,58,59,60,61,62,63,64]. As an endogenous timekeeper for the circadian system, irregular timing of meals may contribute to circadian disruption which could further influence irregular sleep patterns [65]. Furthermore, sleep and dietary habits share common causes in underlying social and environmental factors [66,67], so their effects on chronic diseases may partially mediate the effects of upstream social determinants on health outcomes. This prior evidence suggests that adverse sleep and diet profiles may co-occur more frequently in the population than would be expected if these behaviors were completely independent. Regardless of how or if these behaviors influence one another, our study suggests that improving at least one of these behaviors (sleep regularity or diet quality) is much more beneficial than not improving either.

### 4.1. Strengths and Limitations

Our study has some limitations. First, we cannot rule out residual confounding nor establish causality given the observational design. Second, a self-reported diet may introduce measurement errors, though likely nondifferential concerning CVD risk. Third, our sample size and relatively few events contributed to imprecise estimates with wide 95% CIs. However, few cohorts have comparable information on diet, device-based sleep, and CVD follow-up. Similarly, we lacked power to investigate sex- or ethnicity-specific effects due to small subgroup sizes, though subgroup differences in sleep regularity and diet quality may influence their joint effects on CVD and warrant future investigation. Fourth, we dichotomized sleep regularity and diet quality to assess interpretable joint effects, despite some loss of information compared to continuous variables. However, we also used the continuous measures to evaluate individual effects, multiplicative interaction, and exploratory visual plots. The results using the continuous measures aligned with our main findings. While the cut-points selected for dichotomization were somewhat arbitrary, the exploratory analysis visually displaying the continuous interaction effects (Figures S12 and S13) suggest that the choice of cut-point should not have impacted the findings. The cut-points were chosen to facilitate clinical interpretation (e.g., 1 or 1.5 h for SD sleep timing and duration) to match the previous literature [9,10,28], and to balance the distribution and ensure adequate sample sizes in each group after cross-tabulation. Future studies with larger sample sizes should explore finer categorizations of these exposures in relation to joint CVD effects. Finally, we were unable to investigate if diet quality and sleep regularity are causally related or changes in these behaviors over time since they were measured once near the same time in our study. However, this is a related and important question for future investigations that would provide more context for interpreting their joint effects on CVD risk.

Despite these limitations, this study has several strengths. First, the use of 7-day actigraphy provided an assessment of sleep regularity unbiased by self-report. Second, the dietary assessment was culturally adapted for this multi-ethnic sample. This analysis used a robust, systematic method to evaluate the joint association of diet quality and sleep regularity by estimating HRs in relation to a common referent group to allow for a direct comparison of individual effects comparison of the joint effect to these individual effects, and an estimation of multiplicative and additive interaction. This method reveals a joint effect with substantial public health relevance, given how universal and common these behaviors are and their potential to co-occur in an unhealthy lifestyle. We also explored the joint effect using continuous measures and found a similar pattern that reinforced our primary findings. We controlled for many potential confounders including self-reported physical activity and depressive symptoms and the results were stable, with additional adjustment for traditional CVD risk factors and other sleep characteristics. The MESA cohort represents a diverse sample of older US adults, increasing the generalizability of our findings to the aging US population.

### 4.2. Conclusions

This study is one of the first to provide evidence that the combined effect of poor diet quality and irregular sleep substantially increases CVD risk. The lack of interaction detected addresses previously unanswered questions, as the prior literature has hypothesized that sleep and diet may have interactive effects. Additive or multiplicative synergistic or antagonistic interactions were not detected, indicating that the joint effect closely represents the combined risk of the individual risks of poor diet quality and irregular sleep. These results reinforce the recommendations to achieve a healthy diet and regular sleep patterns, through multi-lifestyle approaches, for cardiovascular risk reduction, especially among those who are affected by a disrupted lifestyle marked jointly by these unhealthy behaviors.

## Figures and Tables

**Table 1:** Description of participants in the total included sample and by regularity of sleep timing and diet quality.

		Sleep Timing Regularity		Diet Quality	
	Total Sample ^a^	Regular ^a^	Irregular ^a^	High ^a^	Low ^a^
n	1782	962	820	930	852
CVD cases/person-years	188/14,323.9	97/7853.1	91/6470.8	88/7579.9	100/6744.0
Rate per 10,000 person-years	131.25	123.52	140.63	116.10	148.28
Age, years	68.2 ± 9.1; 67 [14]	68.7 ± 8.9; 68 [15]	67.6 ± 9.3; 66 [14]	68.6 ± 9.0; 68 [15]	67.7 ± 9.1; 66 [15]
Women	977 (54.8)	536 (55.7)	441 (53.8)	538 (57.9)	439 (51.5)
Race/ethnicity					
White	699 (39.2)	431 (44.8)	268 (32.7)	392 (42.2)	307 (36.0)
Chinese	205 (11.5)	100 (10.4)	105 (12.8)	154 (16.6)	51 (6.0)
Black	480 (26.9)	202 (21.0)	278 (33.9)	196 (21.1)	284 (33.3)
Hispanic/Latino	398 (22.3)	229 (23.8)	169 (20.6)	188 (20.2)	210 (24.7)
Education					
<High school	235 (13.4)	124 (12.9)	111 (13.5)	109 (11.7)	126 (14.8)
High school degree	284 (16.5)	165 (17.2)	119 (14.5)	125 (13.4)	159 (18.7)
Some college	532 (29.9)	280 (29.1)	252 (30.7)	245 (26.3)	287 (33.7)
College degree	731 (41.0)	393 (40.9)	338 (41.2)	451 (48.5)	280 (32.9)
Employed	440 (24.7)	233 (24.2)	207 (25.2)	211 (22.7)	229 (26.9)
Shiftwork	177 (10.1)	79 (8.3)	98 (12.1)	88 (9.6)	89 (10.5)
Married	1093 (61.3)	615 (63.9)	478 (58.3)	575 (61.8)	518 (60.8)
Smoking history (pack-years)	9.9 ± 18.6; 0 [12.6]	9.5 (17.3); 0 [12.9]	10.4 (20); 0 [12.5]	8.4 (16.5); 0 [10.5]	11.5 (20.5); 0 [15]
Smoking status					
Never	845 (47.5)	459 (47.7)	386 (47.1)	467 (50.3)	378 (44.4)
Former	810 (45.5)	452 (47.0)	358 (43.7)	414 (44.6)	396 (46.5)
Current	126 (7.1)	51 (5.3)	75 (9.2)	48 (5.2)	78 (9.2)
Physical activity, MET-h/week	91.3 ± 103.2; 62.1 [89.2]	89.1 ± 87.1; 64.1 [83.5]	93.9 ± 119.4; 59.4 [95.4]	89.9 ± 88.4; 63.7 [88.9]	92.8 ± 117.4; 60.8 [91.1]
Depressive symptoms	252 (14.1)	103 (10.7)	149 (18.2)	114 (12.3)	138 (16.2)
CES-D score	8.1 ± 7.5; 6 [9]	7.4 ± 7.0; 6 [8]	9.0 ± 8.0; 7 [10]	7.5 ± 7.1; 6 [9]	8.9 ± 7.9; 7 [10]
Body Mass Index, kg/m^2^	28.7 ± 5.6; 27.9 [7.3]	28.5 ± 5.4; 27.7 [7.1]	28.9 ± 5.7; 28.3 [7.4]	27.5 ± 5.0; 26.9 [6.6]	30.0 ± 5.9; 29.2 [7.3]
**Sleep**					
Nights of actigraphy	7 ± 0.5; 7 [0]	7 ± 0.4; 7 [0]	7 ± 0.6; 7 [0]	7 ± 0.4; 7 [0]	7.0 ± 0.6; 7 [0]
Season of actigraphy					
Winter (December–February)	238 (13.2)	125 (13.0)	113 (13.8)	115 (12.4)	123 (14.4)
Spring (March–May)	294 (16.5)	169 (17.6)	125 (15.2)	163 (17.5)	131 (15.4)
Summer (June–August)	339 (19.0)	187 (19.4)	152 (18.5)	197 (21.2)	142 (16.7)
Fall (September–November)	911 (51.1)	481 (50.0)	430 (52.4)	455 (48.9)	456 (53.5)
Average sleep duration, hours	6.6 ± 1.2; 6.7 [1.5]	6.9 ± 1.0; 7.0 [1.2]	6.1 ± 1.3; 6.2 [1.7]	6.6 ± 1.2; 6.7 [1.4]	6.5 ± 1.3; 6.6 [1.6]
SD of sleep duration, minutes	75.3 ± 37.5; 68.8 [49.5]	56.2 ± 26.3; 52.3 [31.8]	97.6 ± 36.4; 93.0 [46.3]	74.3 ± 38.3; 68.3 [51.9]	76.3 ± 36.6; 69.6 [48.1]
Irregular sleep duration (SD sleep duration > 90 min)	545 (30.6)	102 (10.6)	443 (54.0)	282 (30.3)	263 (30.9)
SD of sleep onset time, minutes	79.8 ± 93.4; 55.9 [53.6]	35.8 ± 13.5; 35.7 [21.5]	131.6 ± 117.5; 92.1 [53.1]	78.7 ± 95.7; 54.0 [53.9]	81.1 ± 90.9; 57.9 [54.0]
Irregular sleep timing (SD sleep onset > 60 min)	820 (46.0)	0 (0.0)	820 (100.0)	404 (43.4)	416 (48.8)
Chronotype					
Morning	904 (51.3)	528 (55.5)	376 (46.4)	490 (53.6)	414 (48.9)
Intermediate	725 (41.2)	375 (39.4)	350 (43.2)	362 (39.6)	363 (42.9)
Evening	133 (7.6)	49 (5.2)	84 (10.4)	63 (6.9)	70 (8.3)
Insomnia symptoms	617 (35.1)	308 (32.4)	309 (38.3)	314 (34.5)	303 (35.8)
Excessive daytime sleepiness	246 (14.0)	111 (11.6)	135 (16.7)	111 (12.1)	135 (15.9)
**Diet**					
Energy intake, kcal/d	1719.3 ± 809.1; 1567.1 [1027.5]	1659.0 ± 737.3; 1539.0 [889.6]	1790.1 ± 881.1; 1607.0 [1230.9]	1746.8 ± 799.0; 1591.3 [989.6]	1689.4 ± 819.4; 1533.5 [1089.5]
AHEI-2010	59.3 ± 10.8; 58.7 [15.6]	60.0 ± 10.7; 59.7 [15.4]	58.5 ± 11.0; 57.9 [15.5]	67.6 ± 6.7; 66.7 [9.3]	50.1 ± 6.0; 51.3 [8.5]
Low diet quality	852 (47.8)	436 (45.3)	416 (50.7)	0 (0.0)	852 (100.0)

^a.^ Continuous variables are reported as mean ± SD; median [IQR]. Categorical variables are reported as *n* (% among column total). Abbreviations: SD: standard deviation; CVD: cardiovascular disease; MET: metabolic-equivalent of task; CES-D: Centers for Epidemiologic Research Depression Scale; Non-HDL: non-high-density lipoprotein cholesterol; AHEI-2010: Alternate Healthy Eating Index 2010. Definitions of categorized variables: shiftwork: usual work schedule is night shift, split shift, irregular shift/on-call, or rotating shifts. Depressive symptoms: CES-D ≥ 16. Diabetes status based on American Diabetes Association 2003 fasting criteria. Hypertensive: (≥140 mm HG systolic or ≥90 mm HG diastolic blood pressure. Irregular sleep duration: SD sleep duration > 90 min; irregular sleep timing: SD sleep onset timing > 60 min. Chronotype: reduced Horne Osteberg Morningness-Eveningness Questionnaire, <12 classified as evening type, 12 to 17 classified as intermediate, >17 classified as morning type. Insomnia symptoms: Women’s Health Initiative Insomnia Rating Scale (WHIIRS) > 8. Excessive daytime sleepiness: Epworth sleepiness scale > 10. Low diet quality: <median AHEI score (AHEI < 58.14) of total sample with valid diet data.

**Table 2:** Joint distribution and cardiovascular disease (CVD) event rate of high/low diet quality and regular/irregular sleep timing and duration.

		High Diet Quality			Low Diet Quality		
		Frequency (%)	CVD Cases/ Person-Years	Event Rate ^a^	Frequency (%)	CVD Cases/ Person-Years	Event Rate ^a^
**Sleep timing regularity**	**Regular**	526 (29.5)	47/4332.2	108.5	436 (24.5)	50/3521.0	142.0
	**Irregular**	404 (22.7)	41/3247.7	126.2	416 (23.3)	50/3223.0	155.1
**Sleep duration regularity**	**Regular**	648 (36.4)	58/5295.4	109.5	589 (33.1)	67/4783.4	140.1
	**Irregular**	282 (15.8)	30/2284.5	131.3	263 (14.8)	33/1960.5	168.3

Low diet quality: <median AHEI score (AHEI < 58.14) of total sample with valid diet data. Irregular sleep timing: SD sleep onset timing > 60 min. Irregular sleep duration: SD sleep duration > 90 min. ^a^ CVD events per 10,000 person-years.

**Table 3:** Adjusted individual associations between diet quality and sleep regularity measures with incident cardiovascular disease.

		Model 1	Model 2	Model 3
		HR (95% CI)	HR (95% CI)	HR (95% CI)
**Diet quality**				
Continuous	AHEI total score (per SD increase)	0.87 (0.75, 1.02)	0.88 (0.75, 1.04)	0.88 (0.75, 1.04)
Binary	High diet quality (AHEI ≥ median)	Ref.	Ref.	Ref.
	Low quality diet (AHEI < median)	1.38 (1.03, 1.86)	1.36 (1.00, 1.85)	1.35 (0.99, 1.84)
Categorical	Quintile 5 (highest quality diet)	Ref.	Ref.	Ref.
	Quintile 4	0.72 (0.45, 1.13)	0.70 (0.44, 1.11)	0.70 (0.45, 1.12)
	Quintile 3	0.86 (0.55, 1.34)	0.81 (0.52, 1.28)	0.82 (0.52, 1.30)
	Quintile 2	0.84 (0.53, 1.34)	0.82 (0.50, 1.32)	0.82 (0.51, 1.33)
	Quintile 1 (lowest quality diet)	1.43 (0.93, 2.19)	1.38 (0.88, 2.16)	1.35 (0.86, 2.12)
	*p* for trend ^a^	0.117	0.181	0.208
**Sleep timing regularity**				
Continuous	SD sleep onset (per 1 h increase)	1.11 (1.03, 1.20)	1.11 (1.03, 1.20)	1.10 (1.02, 1.19)
Binary	Regular (SD sleep onset < 60 min)	Ref.	Ref.	Ref.
	Irregular (SD sleep onset ≥ 60 min)	1.25 (0.94, 1.67)	1.23 (0.92, 1.65)	1.21 (0.91, 1.63)
Categorical ^b^	SD sleep onset < 30 min	Ref.	Ref.	Ref.
	SD sleep onset 30 to <60 min	0.94 (0.63, 1.42)	0.93 (0.62, 1.41)	0.92 (0.61, 1.40)
	SD sleep onset 60 to <90 min	1.12 (0.73, 1.73)	1.10 (0.71, 1.70)	1.08 (0.70, 1.69)
	SD sleep onset ≥ 90 min	1.29 (0.84, 1.98)	1.28 (0.83, 1.97)	1.24 (0.80, 1.91)
	*p* for trend ^a^	0.144	0.170	0.219
**Sleep duration regularity**				
Continuous ^b^	SD sleep duration (per 1 h increase)	1.26 (1.01, 1.58)	1.25 (1.00, 1.57)	1.24 (0.98, 1.55)
Binary	Regular (SD sleep duration < 90 min)	Ref.	Ref.	Ref.
	Irregular (SD sleep duration ≥ 90 min)	1.28 (0.94, 1.73)	1.27 (0.93, 1.72)	1.25 (0.92, 1.71)
Categorical	SD sleep duration < 60 min	Ref.	Ref.	Ref.
	SD sleep duration 60 to <90 min	1.21 (0.85, 1.74)	1.17 (0.85, 1.68)	1.15 (0.80, 1.66)
	SD sleep duration 90 to <120 min	1.30 (0.87, 1.94)	1.25 (0.84, 1.88)	1.23 (0.82, 1.85)
	SD sleep duration ≥ 120 min	1.55 (0.98, 2.44)	1.54 (0.98, 2.43)	1.52 (0.96, 2.41)
	*p* for trend ^a^	0.046	0.059	0.073

HR: hazard ratio. CI: confidence interval. SD: standard deviation. AHEI: Alternate Healthy Eating Index 2010. SD of AHEI = 10.8 points (the AHEI total score can take values from 0 to 110 points). Low diet quality: <median AHEI score (AHEI < 58.14) of the total sample with valid diet data. ^a^ *p* for trend is calculated by assigning the median AHEI score of each quintile to all subjects in that quintile and then treating this as a continuous variable in the model and reporting the *p*-value associated with this variable’s beta estimate. *p* for trend for SD sleep onset time and for SD sleep duration is calculated by assigning numeric values 0, 1, 2, and 3 to the 4 groups from shortest to longest SD sleep values and treating these as continuous variables in the model and reporting the *p* = value associated with this variable’s estimate. ^b^ Did not meet the proportional hazards assumption based on Schoenfeld tests but were not major violators of this assumption based upon log-log plots. Estimates are from Cox proportional hazards regression models adjusting for the following covariates: Model 1: age, sex, race/ethnicity. Model 2: model 1 + total energy intake (kcal/d; diet models only), height (cm; diet models only), education (<high school, high school degree, some college, and bachelor’s degree or higher), employment status (employed/unemployed), marital status (currently married/single, divorced, and widowed), study site, season of actigraphy recording (sleep models only; winter, spring, summer, and fall). Model 3: model 2 + physical activity (self-report, MET-hours per week), smoking history (pack-years), depressive symptoms (CES-D ≥ 16).

**Table 4:** Adjusted individual and joint hazard ratios for diet quality and sleep regularity for incident total cardiovascular disease, and estimates of multiplicative and additive interaction.

	Diet Quality		
	High (AHEI ≥ Median)	Low (AHEI < Median)	
**Sleep timing regularity**			
			Effect of low (vs. high) diet quality within strata of sleep timing regularity
**Regular** (SD sleep onset time < 60 min)	1.00	1.37 (0.91, 2.08)	1.37 (0.91, 2.08)
**Irregular** (SD sleep onset time ≥ 60 min)	1.24 (0.81, 1.90)	1.56 (1.03, 2.37)	1.26 (0.82, 1.94)
Effect of irregular sleep timing (vs. regular) within strata of diet quality	1.24 (0.81, 1.90)	1.14 (0.76, 1.71)	
Multiplicative interaction ^a^	0.92 (0.51, 1.64)	Interaction-*p* ^b^, using continuous AHEI and SD sleep onset: 0.340	
Additive interaction (RERI) ^c^	−0.05 (−1.00, 0.66)		
**Sleep duration regularity**			
			Effect of low (vs. high) diet quality within strata of sleep duration regularity
**Regular** (SD sleep duration < 90 min)	1.00	1.31 (0.91, 1.90)	1.31 (0.91, 1.90)
**Irregular** (SD sleep duration ≥ 90 min)	1.23 (0.78, 1.92)	1.70 (1.09, 2.67)	1.39 (0.84, 1.99)
Effect of irregular sleep duration (vs. regular) within strata of diet quality	1.23 (0.78, 1.92)	1.30 (0.85, 1.99)	
Multiplicative interaction ^a^	1.06 (0.58, 1.96)	Interaction-*p* ^b^, for continuous AHEI and SD sleep duration: 0.805	
Additive interaction (RERI) ^c^	0.17 (−0.78, 1.04)		

Cells include adjusted hazard ratios (95% confidence intervals) in comparison to the relevant common reference group, except where noted. AHEI: Alternate Healthy Eating Index; SD: standard deviation; RERI: Relative Excess Risk due to Interaction. Estimates are from Cox proportional hazards models adjusted for the following covariates: age, sex, race/ethnicity + total energy intake (kcal/d; diet models only), height (cm; diet models only), education (<high school, high school degree, some college, and bachelor’s degree or higher), employment status (employed/unemployed), marital status (currently married/single, divorced, and widowed), study site, season of actigraphy recording (sleep models only; winter, spring, summer, and fall) + physical activity (self-report, MET-hours per week), smoking history (pack-years), depressive symptoms (CES-D ≥ 16). ^a^ Multiplicative interaction calculated using HRs with the common reference group: $\frac{{HR}_{11}}{{HR}_{10}*{HR}_{01}}$. ^b^ Interaction *p*-value from the product term of the continuous AHEI and SD sleep variables included in the adjusted model. ^c^ Additive interaction calculated using HRs with the common reference group: RERI = ${HR}_{11}-\left({HR}_{10}+{HR}_{01}\right)+1.$

## Data Availability

Data availability: The data used in this study are available from the MESA Coordinating Center. Researchers can apply for data access through the MESA website (www.mesa-nhlbi.org) by following the standardized procedures published on the website. The actigraphy data are also available through the National Sleep Research Resource (sleepdata.org) through a data use agreement. Code availability: All computation central to the conclusions of this analysis was conducted with R version 4.3.0 and the following R packages: survival (https://CRAN.R-project.org/package=survival, accessed on 21 October 2024), ggplot2 (https://CRAN.R-project.org/package=ggplot2, accessed on 21 October 2024), survminer (https://CRAN.R-project.org/package=survminer, accessed on 21 October 2024), interactionR (https://CRAN.R-project.org/package=interactionR, accessed on 21 October 2024), mgcv (https://CRAN.R-project.org/package=mgcv, accessed on 21 October 2024), and cowplot (https://CRAN.R-project.org/package=cowplot, accessed on 21 October 2024).

## Supplementary Materials

The following supporting information can be downloaded at: https://www.mdpi.com/article/10.3390/nu17111750/s1, Figure S1. Spearman correlation plot of main analysis variables among included participants, n = 1782. Figure S2. Distribution of sleep onset time regularity, the intra-individual night-to-night standard deviation of sleep onset time from actigraphy, among included participants, n = 1782. Figure S3. Distribution of sleep midpoint time regularity, the intra-individual night-to-night standard deviation of sleep midpoint time from actigraphy, among included participants, n = 1782. Figure S4. Distribution of sleep duration regularity, the intra-individual night-to-night standard deviation of sleep duration from actigraphy, among included participants, n = 1782. Table S1. Components and scoring of the Alternate Healthy Eating Index 2010 dietary pattern. Figure S5. Distribution of Alternate Healthy Eating Index-2010 at Exam 5 among included participants, n = 1782. Table S2. Description of the presentation of joint associations and interaction results. Figure S6. Survival curves and 95% confidence intervals for incident cardiovascular disease comparing those with high vs. low diet quality, n = 1782. Figure S7. Survival curves and 95% confidence intervals for incident cardiovascular disease comparing quintiles of AHEI score, n = 1782. Figure S8. Survival curves and 95% confidence intervals for incident cardiovascular disease comparing sleep timing regularity groups. Figure S9. Survival curves and 95% confidence intervals for incident cardiovascular disease comparing sleep duration regularity groups. Table S3. Sensitivity models for adjusted individual associations between diet quality and sleep regularity measures with incident cardiovascular disease. Figure S10. Survival curves and 95% confidence intervals of the joint diet quality and sleep timing regularity association with incident CVD. Figure S11. Survival curves and 95% confidence intervals of the joint diet quality and sleep duration regularity association with incident CVD. Table S4. Sensitivity model + shiftwork. Adjusted individual and joint hazard ratios (95% confidence intervals) for incident total cardiovascular disease of sleep timing regularity and diet quality. Table S5. Sensitivity model + other CVD risk factors. Adjusted individual and joint hazard ratios (95% confidence intervals) for incident total cardiovascular disease of sleep timing regularity and diet quality. Table S6. Sensitivity model + other sleep characteristics. Adjusted individual and joint hazard ratios (95% confidence intervals) for incident total cardiovascular disease of sleep timing regularity and diet quality. Table S7. Sensitivity model + shiftwork. Adjusted individual and joint hazard ratios (95% confidence intervals) for incident total cardiovascular disease of sleep duration regularity and diet quality. Table S8. Sensitivity model + CVD risk factors. Adjusted individual and joint hazard ratios (95% confidence intervals) for incident total cardiovascular disease of sleep duration regularity and diet quality. Table S9. Sensitivity model + other sleep characteristics. Adjusted individual and joint hazard ratios (95% confidence intervals) for incident total cardiovascular disease of sleep duration regularity and diet quality. Figure S12. Interaction plot for diet quality and sleep timing regularity. Figure S13. Interaction plot for diet quality and sleep duration regularity.
