# Supplementary material for: The Joint Association of Diet Quality and Sleep Regularity with Incident Cardiovascular Disease in the Multi-Ethnic Study of Atherosclerosis
Source: Nutrients. 2025 May 22;17(11):1750. doi: 10.3390/nu17111750 (PMC12158227; doi:10.3390/nu17111750)
Supplement: Supplementary file 1 [file nutrients-17-01750-s001.zip › nutrients-3624950-supplementary.pdf]

Supplementary Tables and Figures

The joint association of diet quality and sleep regularity with incident cardiovascular disease

Kaitlin S. Potts [1,*,**], Claire Veldkamp [1,*], Tianyi Huang [2], Alexis C. Wood [3], Erin D. Michos [4], Raymond Noordam [5,6] , Susan Redline [1], Heming Wang [1,**]

[1] Brigham and Women's Hospital, Department of Medicine, Division of Sleep and Circadian Disorders, Boston, U.S.A.
[2] Laboratory of Epidemiology and Population Sciences, Intramural Research Program, National Institute on Aging, Baltimore, U.S.A.
[3] USDA/ARS Children's Nutrition Research Center, Baylor College of Medicine, Houston, U.S.A.
[4] Division of Cardiology, Johns Hopkins University School of Medicine, Baltimore, U.S.A.
[5] Department of Internal Medicine, Section of Gerontology and Geriatrics, Leiden University Medical Center, Leiden, The Netherlands
[6] Health Campus The Hague/Department of Public Health and Primary Care, Leiden University Medical Center, The Hague, The Netherlands
[*] These authors contributed equally.
[**] Correspondence: Kaitlin S. Potts, kspotts@bwh.harvard.edu and Heming Wang, hwang@bwh.harvard.edu

List of supplementary tables and figures

Figure S1. Spearman correlation plot of main analysis variables among included participants, n= 1782.
Figure S2. Distribution of sleep onset time regularity, the intra-individual night-to-night standard deviation of sleep onset time from actigraphy, among included participants, n= 1782.
Figure S3. Distribution of sleep midpoint time regularity, the intra-individual night-to-night standard deviation of sleep midpoint time from actigraphy, among included participants, n= 1782.
Figure S4. Distribution of sleep duration regularity, the intra-individual night-to-night standard deviation of sleep duration from actigraphy, among included participants, n= 1782.
Table S1. Components and scoring of the Alternate Healthy Eating Index 2010 dietary pattern.
Figure S5. Distribution of Alternate Healthy Eating Index-2010 at Exam 5 among included participants, n= 1782.
Table S2. Description of the presentation of joint associations and interaction results.
Figure S6. Survival curves and 95% confidence intervals for incident cardiovascular disease comparing those with high vs. low diet quality based on AHEI-2010 score (high diet quality if AHEI>= median, low diet quality if AHEI<median), n=1782.
Figure S7. Survival curves and 95% confidence intervals for incident cardiovascular disease comparing quintiles of AHEI score (diet quality), n=1782.

Figure S8. Survival curves and 95% confidence intervals for incident cardiovascular disease comparing sleep timing regularity groups (60-minute cutoff: SD sleep onset time).
Figure S9. Survival curves and 95% confidence intervals for incident cardiovascular disease comparing sleep duration regularity groups (90-minute cutoff: SD sleep duration).
Table S3. Sensitivity models for adjusted individual associations between diet quality and sleep regularity measures with incident cardiovascular disease.
Figure S10. Survival curves and 95% confidence intervals of the joint diet quality and **sleep timing** regularity association with incident CVD. AHEI ≥ median value; poor diet: AHEI < median values; regular sleep: SD sleep onset time < 60 minutes; irregular sleep: SD sleep onset time ≥ 60 minutes.
Figure S11. Survival curves and 95% confidence intervals of the joint diet quality and **sleep duration** regularity association with incident CVD. Quality diet: AHEI ≥ median value; poor diet: AHEI < median values; regular sleep: SD sleep duration < 90 minutes; irregular sleep: SD sleep duration ≥ 90 minutes.
Table S4. Sensitivity model + shiftwork. Adjusted individual and joint hazard ratios (95% confidence intervals) for incident total cardiovascular disease of **sleep timing regularity** and diet quality.
Table S5. Sensitivity model + other CVD risk factors. Adjusted individual and joint hazard ratios (95% confidence intervals) for incident total cardiovascular disease of **sleep timing regularity** and diet quality.
Table S6. Sensitivity model + other sleep characteristics. Adjusted individual and joint hazard ratios (95% confidence intervals) for incident total cardiovascular disease of **sleep timing regularity** and diet quality.
Table S7. Sensitivity model + shiftwork. Adjusted individual and joint hazard ratios (95% confidence intervals) for incident total cardiovascular disease of **sleep duration regularity** and diet quality.
Table S8. Sensitivity model + CVD risk factors. Adjusted individual and joint hazard ratios (95% confidence intervals) for incident total cardiovascular disease of **sleep duration regularity** and diet quality.
Table S9. Sensitivity model + other sleep characteristics. Adjusted individual and joint hazard ratios (95% confidence intervals) for incident total cardiovascular disease of **sleep duration regularity** and diet quality.
Figure S12. Interaction plot for diet quality and sleep timing regularity.
Figure S13. Interaction plot for diet quality and sleep duration regularity.

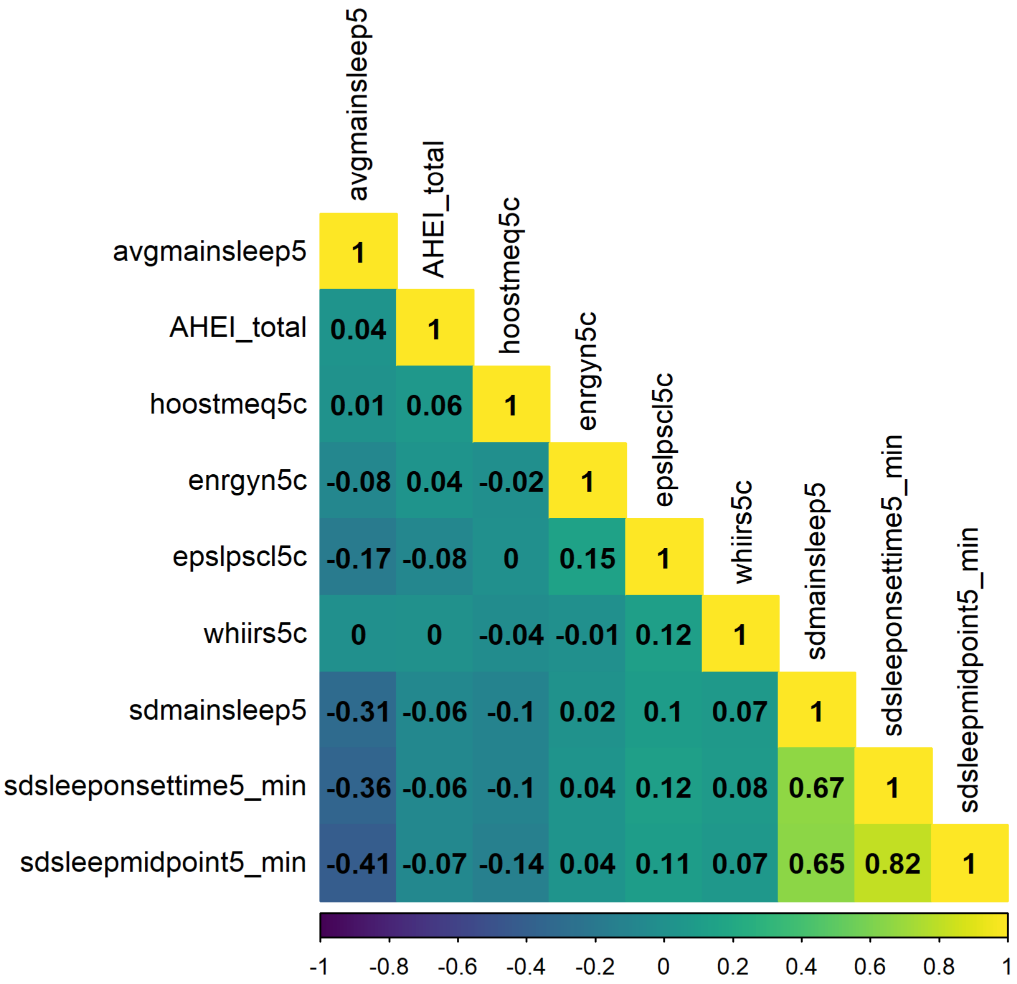

Figure S1. Spearman correlation plot of main analysis variables among included participants, n= 1782.

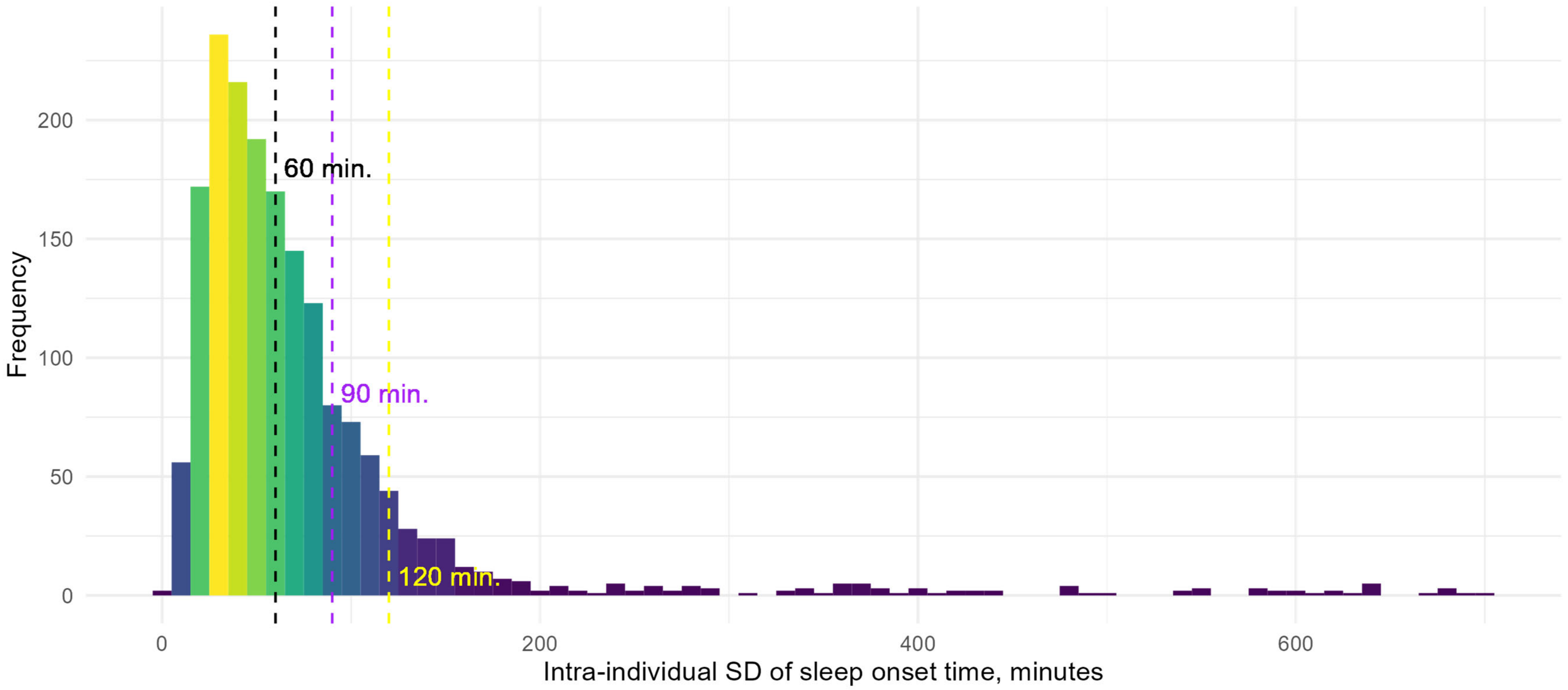

Figure S2. Distribution of sleep onset time regularity, the intra-individual night-to-night standard deviation of sleep onset time from actigraphy among included participants, n= 1782.

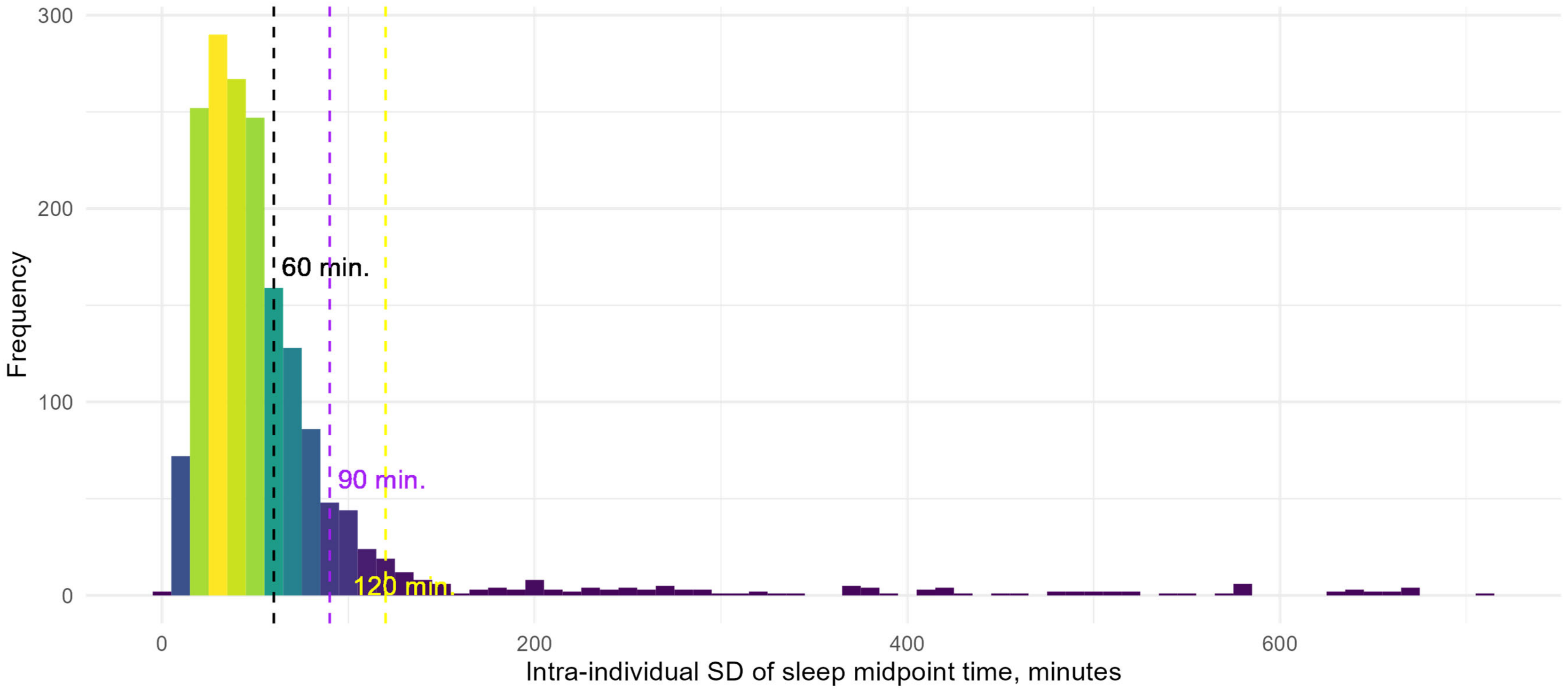

Figure S3. Distribution of sleep midpoint time regularity, the intra-individual night-to-night standard deviation of sleep midpoint time from actigraphy among included participants, n= 1782.

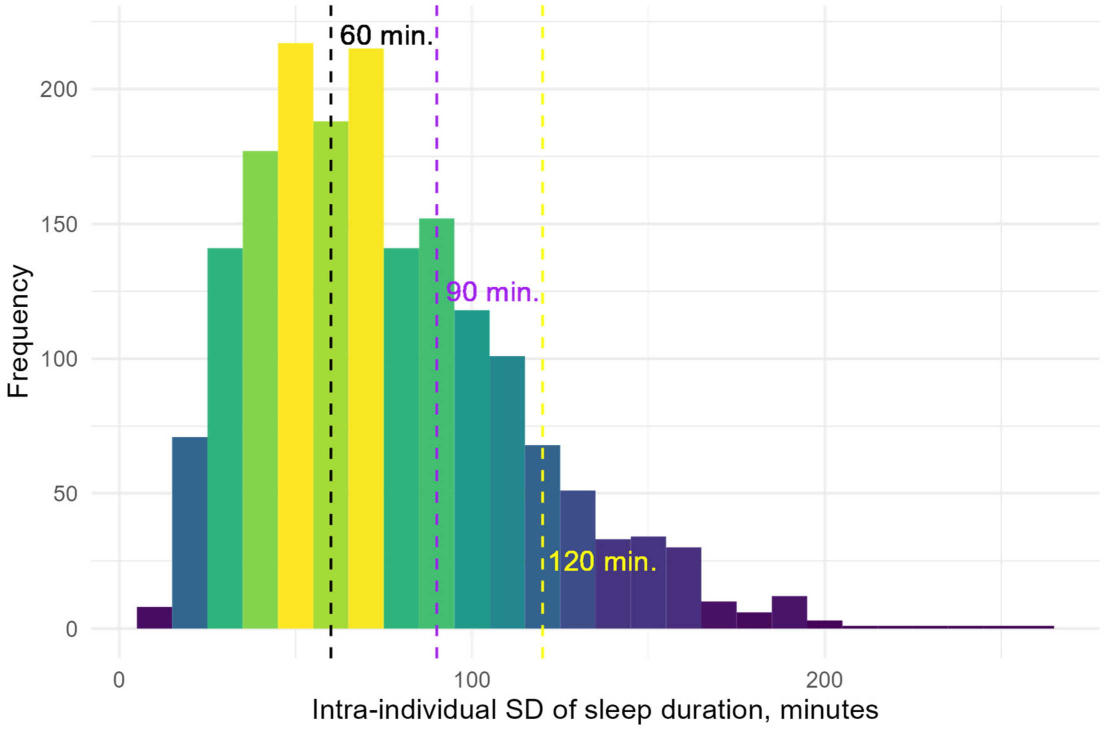

Figure S4. Distribution of sleep duration regularity, the intra-individual night-to-night standard deviation of sleep duration from actigraphy among included participants, n= 1782.

Table S1. Components and scoring of the Alternate Healthy Eating Index 2010 dietary pattern [1].

**Summary**	11 components<br>Total score: 0-110	Components are scored from 0 to 10 based on the daily intake criteria for the minimum (0) and maximum (10) score. Intakes falling between these amounts are scored proportionately from 0 to 10.	
	**Component**	**Scoring criteria for scores of 0 and 10**	**Serving size notes and examples**
Higher intake → higher score	1. Fruits	0: 0 servings<br>10: ≥ 4 servings	1 serving =1 medium piece of fruit or 0.5 cups of berries
	2. Vegetables (not potatoes)	0: 0 servings<br>10: ≥ 5 servings	1 serving = 0.5 cups of vegetables or 1 cup of greens
	3. Nuts and legumes	0: 0 servings<br>10: ≥ 1 servings	1 serving = 1 oz of nuts or 1 tbsp of nut butter
	4. Whole grains	0: 0 grams<br>10: men, ≥ 90 grams; women, ≥ 75 grams	90 grams/day is approximately 6 servings; 75 grams/day is approximately 5 servings
	5. Long chain (n-3) fatty acids (EPA + DHA)	0: 0 mg<br>10: ≥ 250 mg	250 mg/day n-3 fats is approximately equivalent to eating 2-4 servings of fish per week
	6. PUFAs	0: ≤ 2% of energy<br>10: ≥ 10% of energy	Based on each individual's total energy intake.
Lower intake → higher score	7. Sugar sweetened beverages and fruit juice	0: 0<br>10: ≥ 1 serving	1 serving = 8 oz beverage
	8. Red and processed meats	0: ≥ 1.5 servings<br>10: 0 servings	1 serving = 4 oz of unprocessed meat or 1.5 oz of processed meat
	9. trans fats	0: ≥ 4% of energy<br>10: ≤ 0.5% of energy	Based on each individual's total energy intake.
	10. Sodium	0: highest decile<br>10: lowest decile	This is the only component based on quantile distributions within the sample to account for food frequency questionnaire's inability to accurately estimate absolute intakes of sodium (e.g., due to the lack of brand specificity in recording processed foods).
Moderate intake → highest score	11. Alcohol	0: men, ≥ 3.5 drinks; women, ≥ 2.5 drinks<br>10: men, 0.5-2.0 drinks; women, 0.5-1.5 drinks	1 drink = 4 oz of wine, 12 oz of beer, or 1.5 oz of liquor

All amounts are intakes per day unless otherwise specified. PUFA: polyunsaturated fatty acids. MUFA: monounsaturated fatty acids. SFA: saturated fatty acids. 1 oz = 28.35 grams; 1 cup of fruit = 236.59 grams.

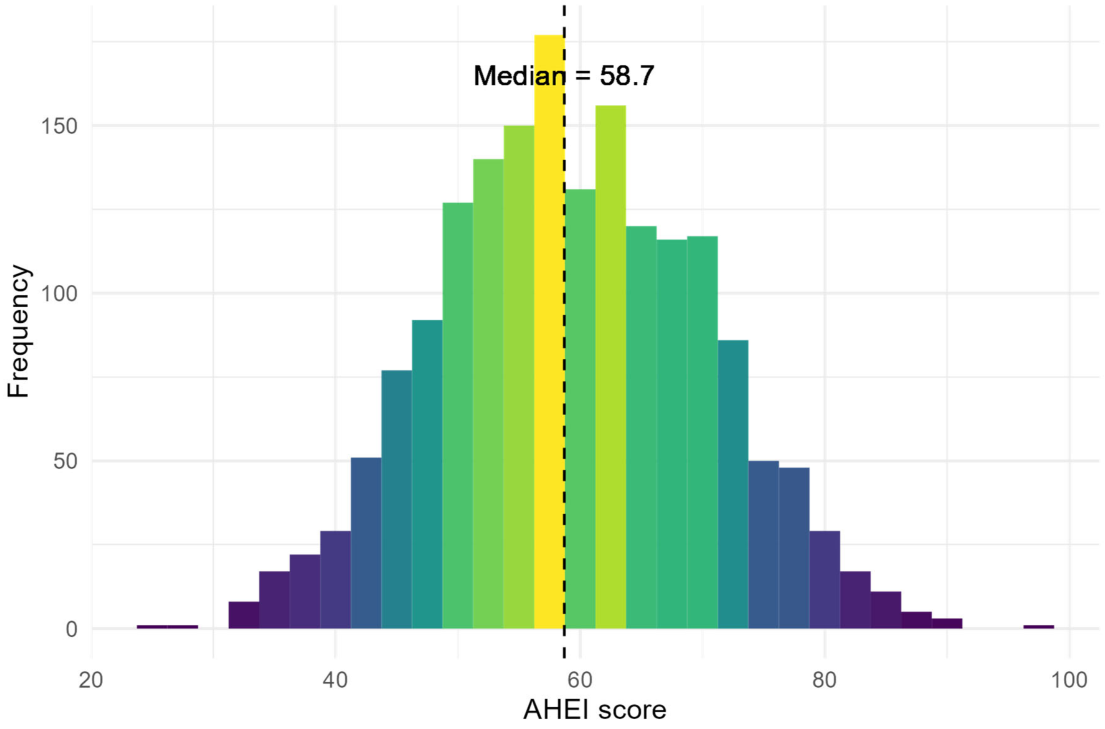

Figure S5. Distribution of Alternate Healthy Eating Index-2010 at Exam 5 among included participants, n= 1782.

Table S2. Description of the presentation of joint associations and interaction results.

		Diet quality		Effect of low-quality diet (vs. high) within strata of sleep regularity
		**High**	**Low**	
**Sleep regularity**	**Regular**	Ref. (HR_{00})	$HR_{01} = HR_{S=0\ D=1\ vs.\ S=0,\ D=0}$	Effect of diet quality with irregular sleep: $HR_{S=0\ D=1\ vs.\ S=0,\ D=0}$
	**Irregular**	$HR_{10} = HR_{S=1,\ D=0\ vs.\ S=0,\ D=0}$	$HR_{11} = HR_{S=1\ D=1\ vs.\ S=0,\ D=0}$	Effect of diet quality with regular sleep: $HR_{S=1,D=1\ vs.\ S=1,\ D=0}$
**Effect of irregular sleep (vs. regular) within strata of diet quality**		Effect of sleep regularity with low diet quality: $HR_{S=1,\ D=0\ vs.\ S=0,\ D=0}$	Effect of sleep regularity with high diet quality: $HR_{S=1,\ D=1\ vs.\ S=0,\ D=1}$	
**Multiplicative interaction**		$\frac{HR_{11}}{HR_{10} * HR_{01}}$	Interaction-p for continuous variables (from the product term of continuous diet quality and continuous sleep regularity variables)	
**Additive interaction (RERI)**		$HR_{11} - (HR_{10} + HR_{01}) + 1$		

This table provides a description of the presentation of the joint results and how to interpret each cell, in following the recommendations by Vanderweele and Knol [2]. The cells include the HRs using the common reference, the stratum-specific results within strata of diet quality and sleep regularity, and the formulas for calculating the measures of multiplicative and additive interaction. S indicates the sleep regularity binary variable where 0 indicates regular sleep and 1 indicates irregular sleep. D indicates the diet quality binary variable, where 0 indicates high diet quality and 1 indicates low diet quality. RERI: Relative Excess Risk due to Interaction.

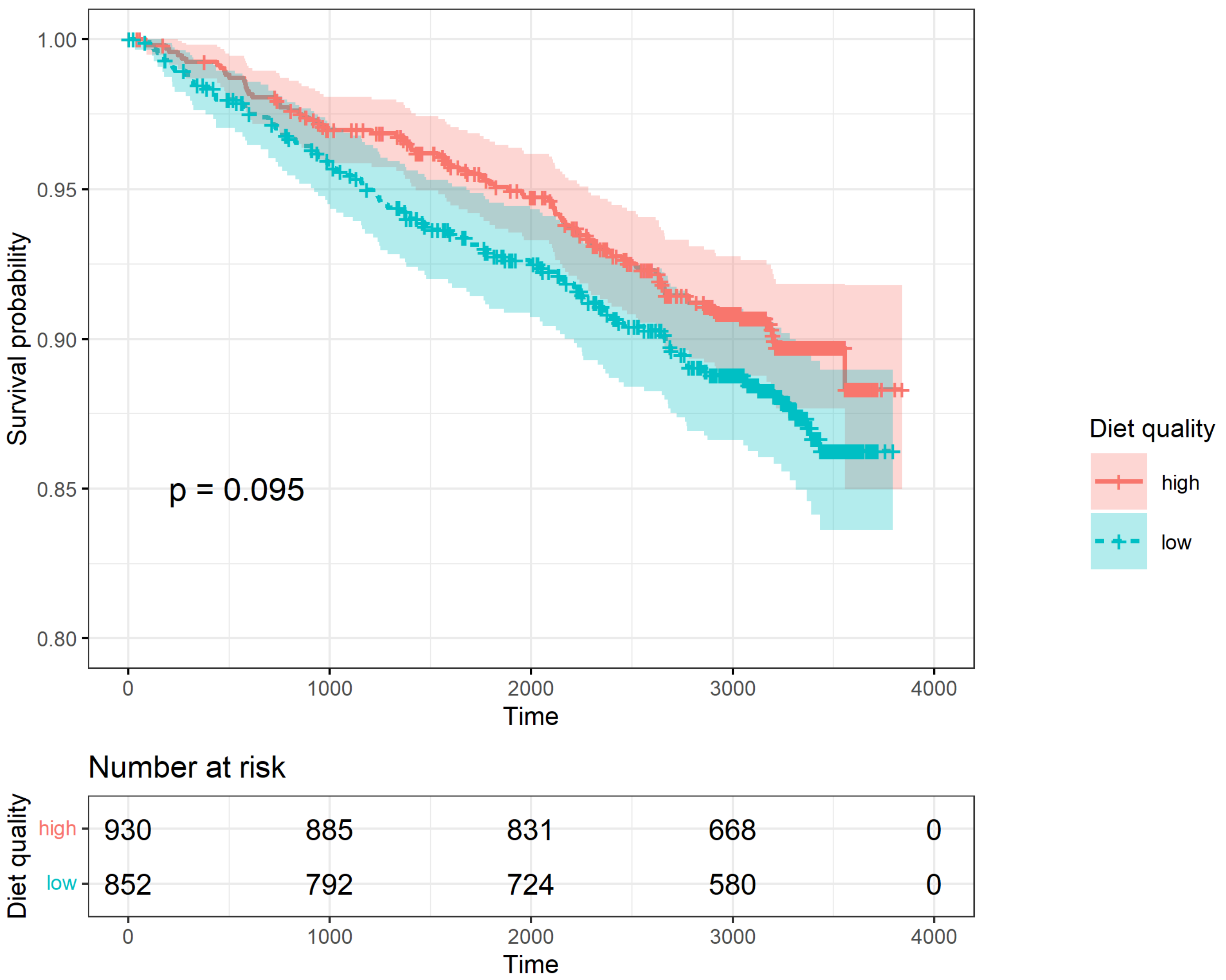

Figure S6. Survival curves and 95% confidence intervals for incident cardiovascular disease comparing those with high vs. low diet quality based on AHEI-2010 score (high diet quality if AHEI>= median, low diet quality if AHEI<median), n=1782.

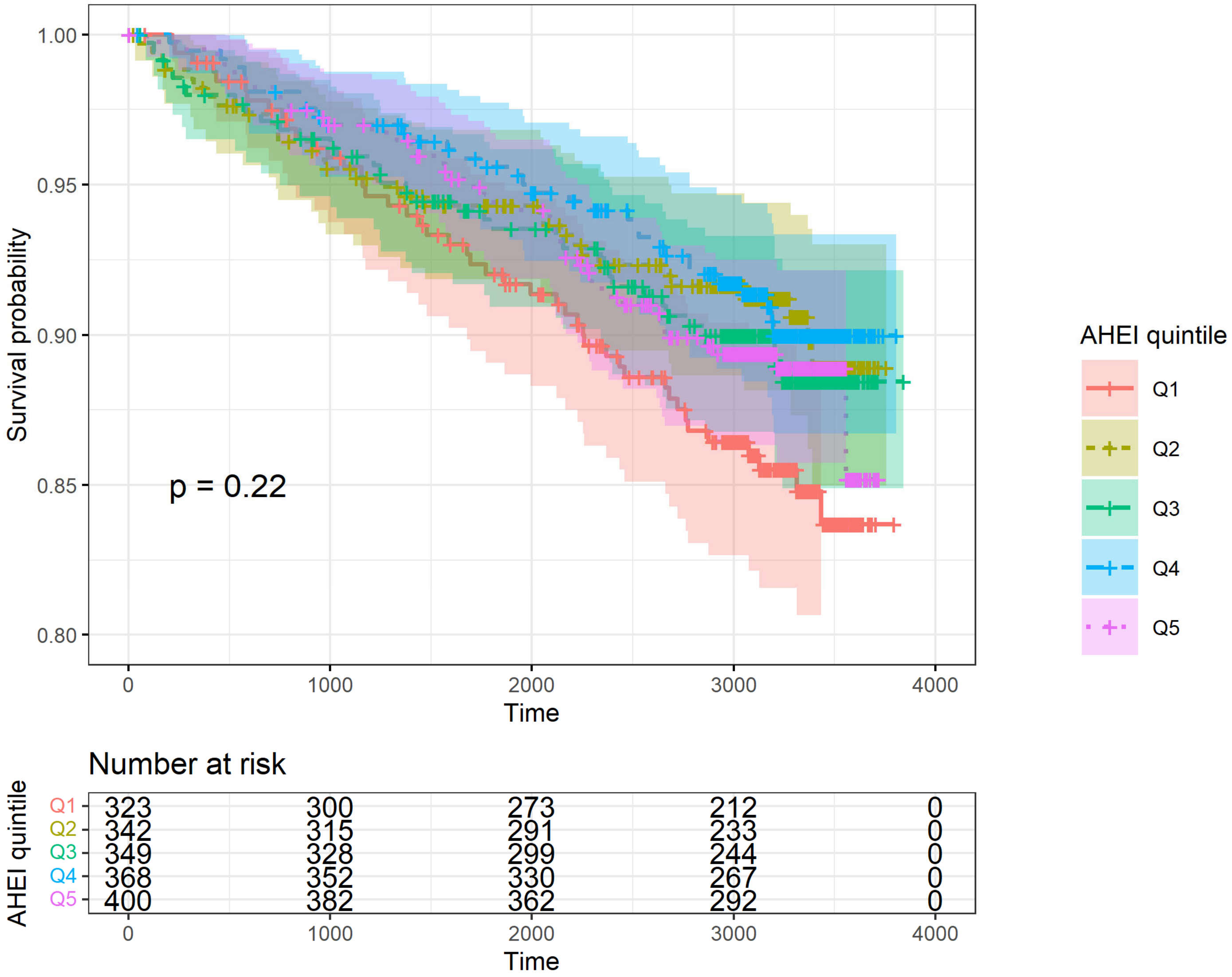

Figure S7. Survival curves and 95% confidence intervals for incident cardiovascular disease comparing quintiles of AHEI score (diet quality), n=1782.

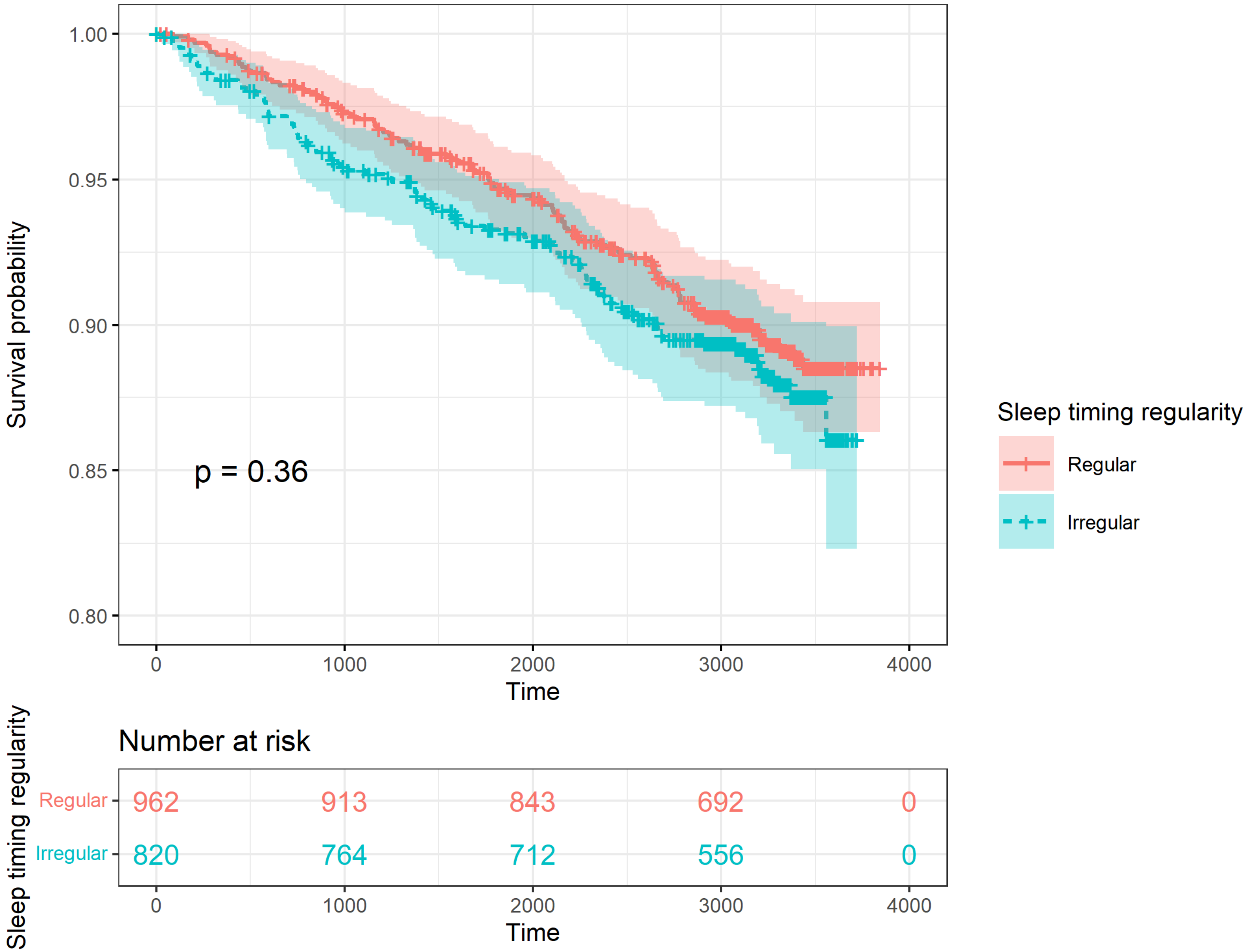

Figure S8. Survival curves and 95% confidence intervals for incident cardiovascular disease comparing sleep timing regularity groups (60-minute cutoff: SD sleep onset time).

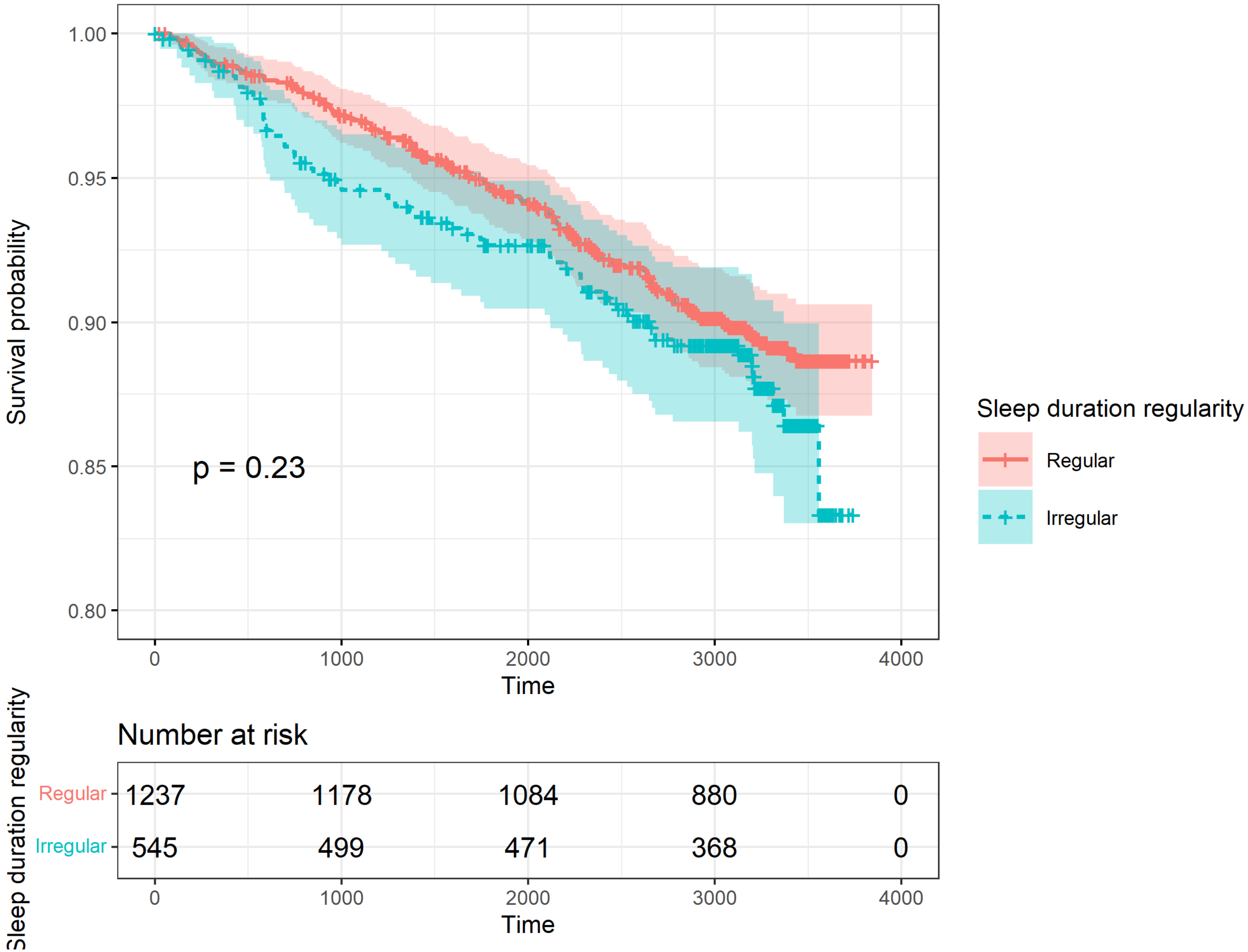

Figure S9. Survival curves and 95% confidence intervals for incident cardiovascular disease comparing sleep duration regularity groups (90-minute cutoff: SD sleep duration).

Table S3. Sensitivity models for adjusted individual associations between diet quality and sleep regularity measures with incident cardiovascular disease.

		Model 3<br>HR (95% CI)	+ shiftwork<br>HR (95% CI)	+ CVD risk factors<br>HR (95% CI)	+ other sleep characteristics<br>HR (95% CI)
**Diet quality**					
Continuous	AHEI total score (per SD increase)	0.88 (0.75, 1.04)	NA	0.90 (0.76, 1.06)	NA
Binary	High diet quality (AHEI ≥ median)	Ref.	NA	Ref.	NA
	Low-quality diet (AHEI < median)	1.35 (0.99, 1.84)	NA	1.36 (0.99, 1.86)	NA
Categorical	Quintile 5 (highest quality diet)	Ref.	NA	Ref.	NA
	Quintile 4	0.70 (0.45, 1.12)	NA	0.71 (0.45, 1.13)	NA
	Quintile 3	0.82 (0.52, 1.30)	NA	0.86 (0.54, 1.36)	NA
	Quintile 2	0.82 (0.51, 1.33)	NA	0.87 (0.53, 1.42)	NA
	Quintile 1 (lowest quality diet)	1.35 (0.86, 2.12)	NA	1.30 (0.81, 2.08)	NA
	p for trend [a]	0.208	NA	0.254	NA
**Sleep timing regularity**					
Continuous	SD sleep onset (per 1 hour increase)	1.10 (1.02, 1.19)	1.10 (1.02, 1.19)	1.11 (1.03, 1.20)	1.10 (1.02, 1.20)
Binary	Regular (SD sleep onset < 60 min.)	Ref.	Ref.	Ref.	Ref.
	Irregular (SD sleep onset ≥ 60 min.)	1.21 (0.91, 1.63)	1.21 (0.90, 1.62)	1.19 (0.88, 1.60)	1.23 (0.90, 1.68)
Categorical	SD sleep onset < 30 min.	Ref.	Ref.	Ref.	Ref.
	SD sleep onset 30 to < 60 min.	0.92 (0.61, 1.40)	0.92 (0.61, 1.40)	0.94 (0.61, 1.43)	0.92 (0.61, 1.40)
	SD sleep onset 60 to < 90 min.	1.08 (0.70, 1.69)	1.10 (0.71, 1.71)	1.06 (0.67, 1.67)	1.12 (0.72, 1.75)
	SD sleep onset ≥ 90 min.	1.24 (0.80, 1.91)	1.20 (0.76, 1.87)	1.22 (0.78, 1.91)	1.23 (0.77, 1.95)
	p for trend [a]	0.219	0.269	0.272	0.257
**Sleep duration regularity**					
Continuous	SD sleep duration (per 1 hour increase)	1.24 (0.98, 1.55)	1.24 (0.99, 1.56)	1.21 (0.96, 1.53)	1.25 (0.99, 1.59)
Binary	Regular (SD sleep duration < 90 min.)	Ref.	Ref.	Ref.	Ref.
	Irregular (SD sleep duration ≥ 90 min.)	1.25 (0.92, 1.71)	1.24 (0.91, 1.70)	1.21 (0.88, 1.66)	1.24 (0.90, 1.72)
Categorical	SD sleep duration < 60 min.	Ref.	Ref.	Ref.	Ref.
	SD sleep duration 60 to < 90 min.	1.15 (0.80, 1.66)	1.18 (0.82, 1.69)	1.14 (0.79, 1.65)	1.21 (0.84, 1.75)
	SD sleep duration 90 to < 120 min.	1.23 (0.82, 1.85)	1.20 (0.79, 1.82)	1.16 (0.77, 1.78)	1.26 (0.83, 1.93)
	SD sleep duration ≥ 120 min.	1.52 (0.96, 2.41)	1.56 (0.99, 2.47)	1.48 (0.93, 2.36)	1.53 (0.94, 2.49)
	p for trend [a]	0.073	0.071	0.115	0.079

HR: hazard ratio. CI: confidence interval. SD: standard deviation. AHEI: Alternate Healthy Eating Index 2010. SD of AHEI = 10.8 points (AHEI total score can take values from 0 to 110 points). Low diet quality: < median AHEI score (AHEI<58.14) of total sample with valid diet data.

[a] p for trend is calculated by assigning the median AHEI score of each quintile to all subjects in that quintile and then treating this as a continuous variable in the model and reporting the p-value associated with this variable's beta estimate. P for trend for SD sleep onset time and for SD sleep duration is calculated by assigning numeric values 0, 1, 2, and 3 to the 4 groups from shortest to longest SD sleep values and treating these as continuous variables in the model and reporting the p=value associated with this variable's estimate.

[b] Did not meet the proportional hazards assumption based on Schenfeld tests but were not major violators of this assumption based upon log-log plots.

Estimates are from Cox proportional hazards regression models adjusting for the following covariates: Model 3: age, sex, race/ethnicity + total energy intake (kcal/d; diet models only), height (cm; diet models only), education (<high school, high school degree, some college, and bachelor's degree or higher), employment status (employed / unemployed), marital status (currently married / single, divorced, and widowed), study site, season of actigraphy recording (sleep models only; winter, spring, summer, and fall) + physical activity (self-report, MET-hours per week), smoking history (pack-years), depressive symptoms (CES-D≥16). Shiftwork: usual work schedule is night shift, split shift, irregular shift/on-call, or rotating shifts. CVD risk factors: BMI, HbA1c, non-HDL cholesterol, and systolic blood pressure. Other sleep characteristics: insomnia symptoms, chronotype, excessive daytime sleepiness, and average sleep duration.

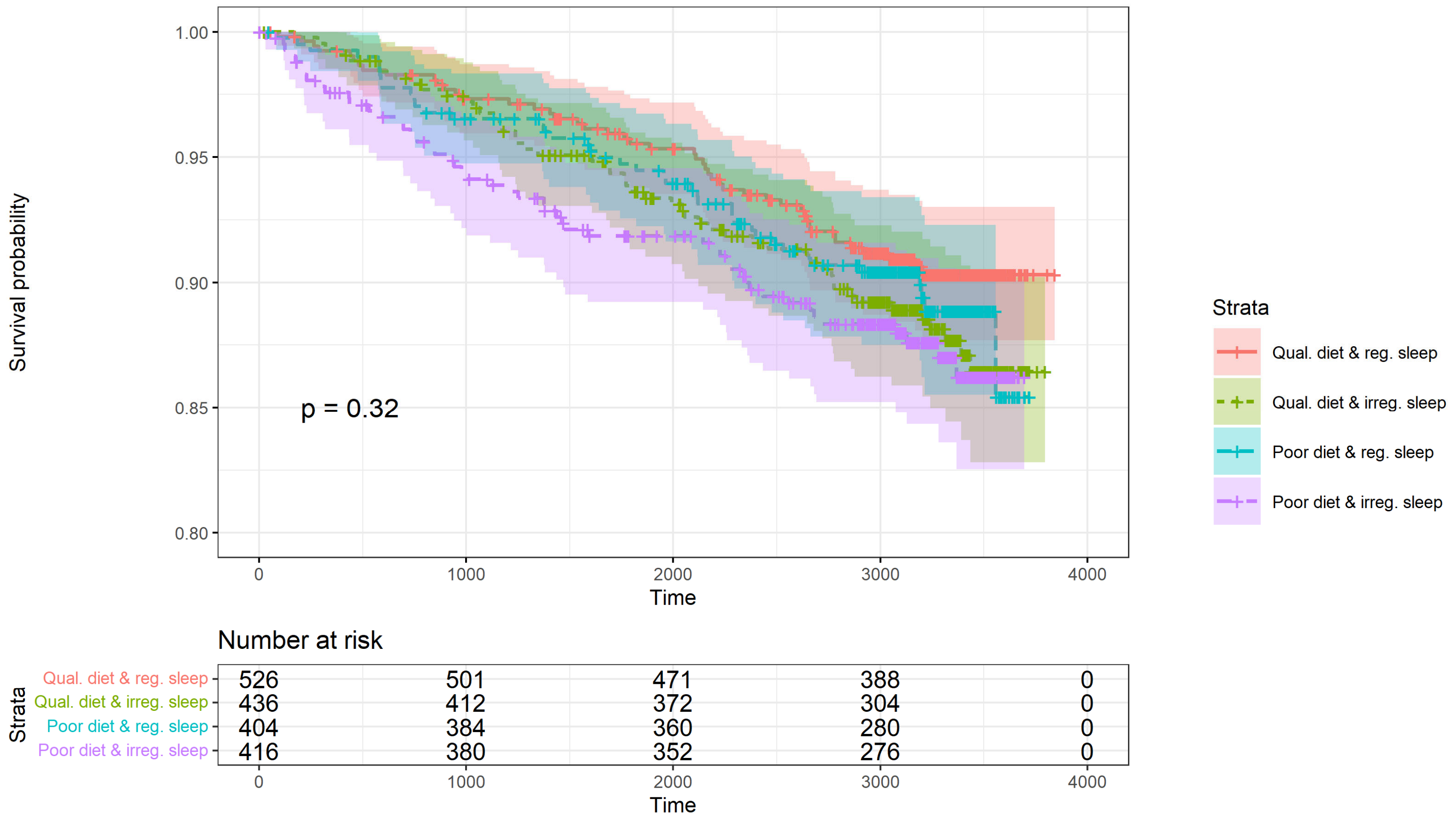

Figure S10. Survival curves and 95% confidence intervals of the joint diet quality and **sleep timing** regularity association with incident CVD. AHEI ≥ median value; poor diet: AHEI < median values; regular sleep: SD sleep onset time < 60 minutes; irregular sleep: SD sleep onset time ≥ 60 minutes.

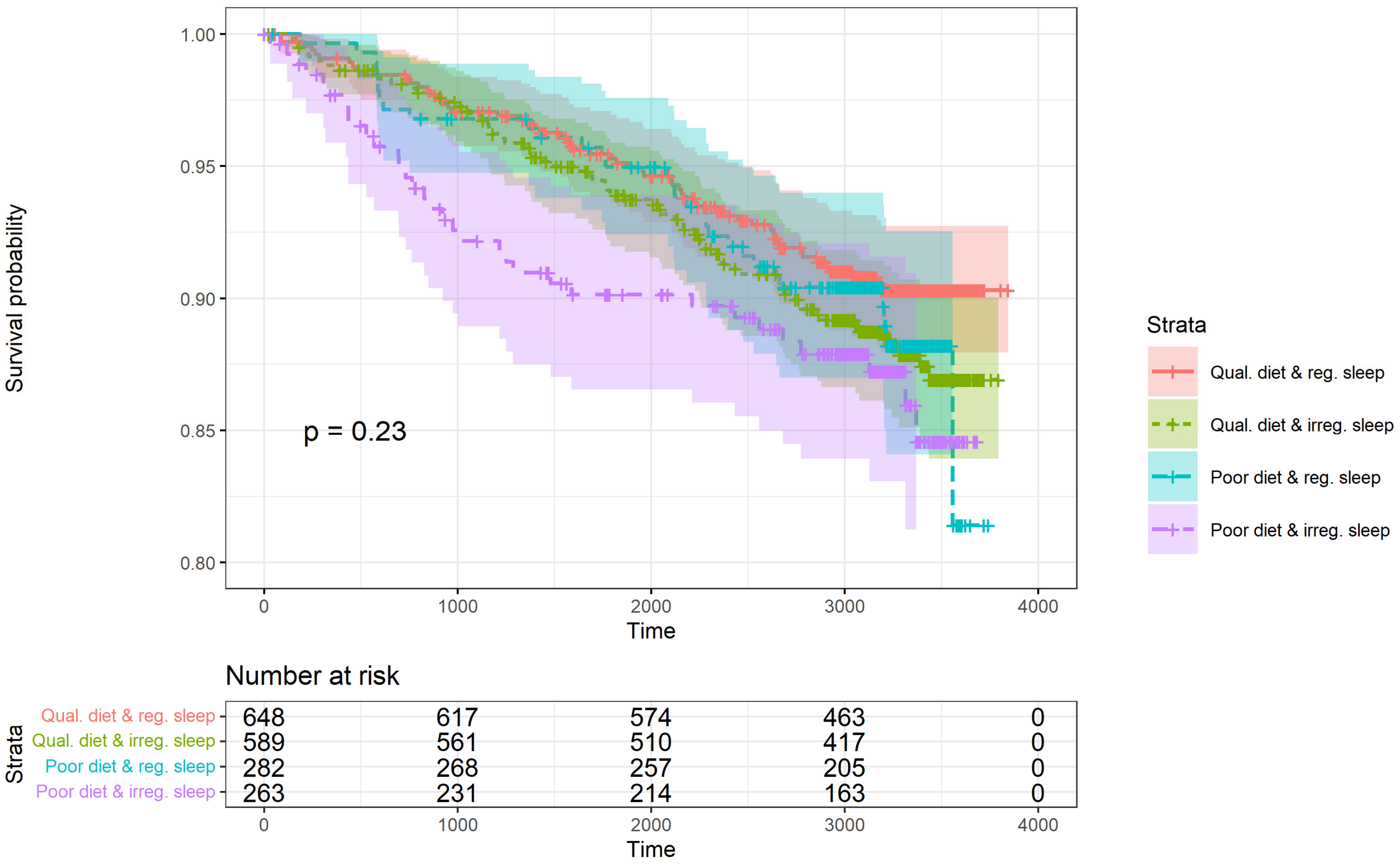

Figure S11. Survival curves and 95% confidence intervals of the joint diet quality and **sleep duration** regularity association with incident CVD. Quality diet: AHEI ≥ median value; poor diet: AHEI < median values; regular sleep: SD sleep duration < 90 minutes; irregular sleep: SD sleep duration ≥ 90 minutes.

Table S4. Sensitivity model + shiftwork. Adjusted individual and joint hazard ratios (95% confidence intervals) for incident total cardiovascular disease of **sleep timing regularity** and diet quality.

		**Diet quality**		**Effect of low-quality diet (vs. high) within strata of sleep regularity**
		**High**	**Low**	
**Sleep regularity**	**Regular**	1.00	1.37 (0.90, 2.08)	1.37 (0.90, 2.08)
	**Irregular**	1.24 (0.80, 1.90)	1.54 (1.01, 2.35)	1.25 (0.81, 1.92)
**Effect of irregular sleep (vs. regular) within strata of diet quality**		1.24 (0.80, 1.90)	1.13 (0.75, 1.69)	
**Multiplicative interaction**		0.91 (0.51, 1.63)	Interaction-p for continuous variables: 0.330	
**Additive interaction (RERI)**		-0.06 (-1.02, 0.65)		

Regular sleep timing regularity: SD sleep onset time < 60 minutes; irregular sleep timing: SD sleep onset time >= 60 minutes; high diet quality: AHEI-2010 >= median value; low-quality diet: AHEI-2010 < median value. Model adjusted for age, sex, race/ethnicity + total energy intake (kcal/d; diet models only), height (cm; diet models only), education (<high school, high school degree, some college, and bachelor's degree or higher), employment status (employed / unemployed), marital status (currently married / single, divorced, and widowed), study site, season of actigraphy recording (sleep models only; winter, spring, summer, and fall) + physical activity (self-report, MET-hours per week), smoking history (pack-years), depressive symptoms (CES-D≥16) + shiftwork (usual work schedule is night shift, split shift, irregular shift/on-call, or rotating shifts).

Multiplicative interaction calculated using HRs with the common reference group: $\frac{HR_{11}}{HR_{10} * HR_{01}}$

Additive interaction calculated using HRs with the common reference group: $HR_{11} - (HR_{10} + HR_{01}) + 1$

Table S5. Sensitivity model + other CVD risk factors. Adjusted individual and joint hazard ratios (95% confidence intervals) for incident total cardiovascular disease of **sleep timing regularity** and diet quality.

		**Diet quality**		**Effect of low-quality diet (vs. high) within strata of sleep regularity**
		**High**	**Low**	
**Sleep regularity**	**Regular**	1.00	1.36 (0.89, 2.08)	1.36 (0.89, 2.08)
	**Irregular**	1.19 (0.77, 1.84)	1.53 (1.00, 2.35)	1.29 (0.83, 2.00)
**Effect of irregular sleep (vs. regular) within strata of diet quality**		1.19 (0.77, 1.84)	1.13 (0.75, 1.70)	
**Multiplicative interaction**		0.95 (0.52, 1.71)	Interaction-p for continuous variables: 0.291	
**Additive interaction (RERI)**		-0.12 (-0.97, 0.70)		

Regular sleep timing regularity: SD sleep onset time < 60 minutes; irregular sleep timing: SD sleep onset time >= 60 minutes; high diet quality: AHEI-2010 >= median value; low-quality diet: AHEI-2010 < median value. Model adjusted for age, sex, race/ethnicity + total energy intake (kcal/d; diet models only), height (cm; diet models only), education (<high school, high school degree, some college, and bachelor's degree or higher), employment status (employed / unemployed), marital status (currently married / single, divorced, and widowed), study site, season of actigraphy recording (sleep models only; winter, spring, summer, and fall) + physical activity (self-report, MET-hours per week), smoking history (pack-years), depressive symptoms (CES-D≥16) + CVD risk factors: BMI, HbA1c, non-HDL cholesterol, and systolic blood pressure.

Multiplicative interaction calculated using HRs with the common reference group: $\frac{HR_{11}}{HR_{10}*HR_{01}}$

Additive interaction calculated using HRs with the common reference group: $HR_{11} - (HR_{10} + HR_{01}) + 1$

Table S6. Sensitivity model + other sleep characteristics. Adjusted individual and joint hazard ratios (95% confidence intervals) for incident total cardiovascular disease of **sleep timing regularity** and diet quality.

		**Diet quality**		**Effect of low-quality diet (vs. high) within strata of sleep regularity**
		**High**	**Low**	
**Sleep regularity**	**Regular**	1.00	1.35 (0.89, 2.05)	1.35 (0.89, 2.05)
	**Irregular**	1.23 (0.79, 1.91)	1.58 (1.03, 2.42)	1.29 (0.83, 1.99)
**Effect of irregular sleep (vs. regular) within strata of diet quality**		1.23 (0.79, 1.91)	1.17 (0.77, 1.77)	
**Multiplicative interaction**		0.95 (0.53, 1.71)	Interaction-p for continuous variables: 0.318	
**Additive interaction (RERI)**		0.00 (-0.63, 0.40)		

Regular sleep timing regularity: SD sleep onset time < 60 minutes; irregular sleep timing: SD sleep onset time >= 60 minutes; high diet quality: AHEI-2010 >= median value; low-quality diet: AHEI-2010 < median value. Model adjusted for age, sex, race/ethnicity + total energy intake (kcal/d; diet models only), height (cm; diet models only), education (<high school, high school degree, some college, and bachelor's degree or higher), employment status (employed / unemployed), marital status (currently married / single, divorced, and widowed), study site, season of actigraphy recording (sleep models only; winter, spring, summer, and fall) + physical activity (self-report, MET-hours per week), smoking history (pack-years), depressive symptoms (CES-D≥16) + other sleep characteristics: insomnia symptoms, chronotype, excessive daytime sleepiness, and average sleep duration.

Multiplicative interaction calculated using HRs with the common reference group: $\frac{HR_{11}}{HR_{10} * HR_{01}}$

Additive interaction calculated using HRs with the common reference group: $HR_{11} - (HR_{10} + HR_{01}) + 1$

Table S7. Sensitivity model + shiftwork. Adjusted individual and joint hazard ratios (95% confidence intervals) for incident total cardiovascular disease of **sleep duration regularity** and diet quality.

		Diet quality		
		**High**	**Low**	**Effect of low-quality diet (vs. high) within strata of sleep regularity**
**Sleep regularity**	**Regular**	1.00	1.30 (0.90, 1.89)	1.30 (0.90, 1.89)
	**Irregular**	1.21 (0.77, 1.91)	1.67 (1.07, 2.63)	1.38 (0.83, 2.31)
**Effect of irregular sleep (vs. regular) within strata of diet quality**		1.21 (0.77, 1.91)	1.28 (0.84, 1.97)	
**Multiplicative interaction**		1.06 (0.57, 1.97)	Interaction-p for continuous variables: p=0.872	
**Additive interaction (RERI)**		0.09 (-0.58, 0.46)		

Regular sleep duration: SD sleep duration < 90 minutes; irregular sleep duration: SD sleep duration >= 90 minutes; high diet quality: AHEI-2010 >= median value; low-quality diet: AHEI-2010 < median value. Model adjusted for age, sex, race/ethnicity + total energy intake (kcal/d; diet models only), height (cm; diet models only), education (<high school, high school degree, some college, and bachelor's degree or higher), employment status (employed / unemployed), marital status (currently married / single, divorced, and widowed), study site, season of actigraphy recording (sleep models only; winter, spring, summer, and fall) + physical activity (self-report, MET-hours per week), smoking history (pack-years), depressive symptoms (CES-D≥16) + shiftwork (usual work schedule is night shift, split shift, irregular shift/on-call, or rotating shifts).

Multiplicative interaction calculated using HRs with the common reference group: $\frac{HR_{11}}{HR_{10} * HR_{01}}$

Additive interaction calculated using HRs with the common reference group: $HR_{11} - (HR_{10} + HR_{01}) + 1$

Table S8. Sensitivity model <u>+ CVD risk factors</u>. Adjusted individual and joint hazard ratios (95% confidence intervals) for incident total cardiovascular disease of **sleep <u>duration</u> regularity** and diet quality.

		Diet quality		
		**High**	**Low**	**Effect of low-quality diet (vs. high) within strata of sleep regularity**
**Sleep regularity**	**Regular**	1.00	1.27 (0.87, 1.86)	1.27 (0.87, 1.86)
	**Irregular**	1.12 (0.71, 1.79)	1.68 (1.07, 2.64)	1.50 (0.89, 2.52)
**Effect of irregular sleep (vs. regular) within strata of diet quality**		1.12 (0.71, 1.79)	1.32 (0.86, 2.03)	
**Multiplicative interaction**		1.18 (0.63, 2.20)	Interaction-p for continuous variables: p=0.609	
**Additive interaction (RERI)**		0.29 (-0.63, 1.15)		

Regular sleep duration: SD sleep duration < 90 minutes; irregular sleep duration: SD sleep duration >= 90 minutes; high diet quality: AHEI-2010 >= median value; low-quality diet: AHEI-2010 < median value. Model adjusted for age, sex, race/ethnicity + total energy intake (kcal/d; diet models only), height (cm; diet models only), education (<high school, high school degree, some college, and bachelor's degree or higher), employment status (employed / unemployed), marital status (currently married / single, divorced, and widowed), study site, season of actigraphy recording (sleep models only; winter, spring, summer, and fall) + physical activity (self-report, MET-hours per week), smoking history (pack-years), depressive symptoms (CES-D≥16) + CVD risk factors: BMI, HbA1c, non-HDL cholesterol, and systolic blood pressure.

Multiplicative interaction calculated using HRs with the common reference group: $\frac{HR_{11}}{HR_{10} * HR_{01}}$

Additive interaction calculated using HRs with the common reference group: $HR_{11} - (HR_{10} + HR_{01}) + 1$

Table S9. Sensitivity model + other sleep characteristics. Adjusted individual and joint hazard ratios (95% confidence intervals) for incident total cardiovascular disease of **sleep duration regularity** and diet quality.

		**Diet quality**		
		**High**	**Low**	**Effect of low-quality diet (vs. high) within strata of sleep regularity**
**Sleep regularity**	**Regular**	1.00	1.29 (0.89, 1.88)	1.29 (0.89, 1.88)
	**Irregular**	1.17 (0.74, 1.87)	1.70 (1.07, 2.69)	1.45 (0.86, 2.43)
**Effect of irregular sleep (vs. regular) within strata of diet quality**		1.17 (0.74, 1.87)	1.32 (0.85, 2.04)	
**Multiplicative interaction**		1.12 (0.60, 2.09)	Interaction-p for continuous variables: p=0.638	
**Additive interaction (RERI)**		0.23 (-0.71, 1.12)		

Regular sleep duration: SD sleep duration < 90 minutes; irregular sleep duration: SD sleep duration >= 90 minutes; high diet quality: AHEI-2010 >= median value; low-quality diet: AHEI-2010 < median value. Model adjusted for age, sex, race/ethnicity + total energy intake (kcal/d; diet models only), height (cm; diet models only), education (<high school, high school degree, some college, and bachelor's degree or higher), employment status (employed / unemployed), marital status (currently married / single, divorced, and widowed), study site, season of actigraphy recording (sleep models only; winter, spring, summer, and fall) + physical activity (self-report, MET-hours per week), smoking history (pack-years), depressive symptoms (CES-D≥16) + other sleep characteristics: insomnia symptoms, chronotype, excessive daytime sleepiness, and average sleep duration.

Multiplicative interaction calculated using HRs with the common reference group: $\frac{HR_{11}}{HR_{10} * HR_{01}}$

Additive interaction calculated using HRs with the common reference group: $HR_{11} - (HR_{10} + HR_{01}) + 1$

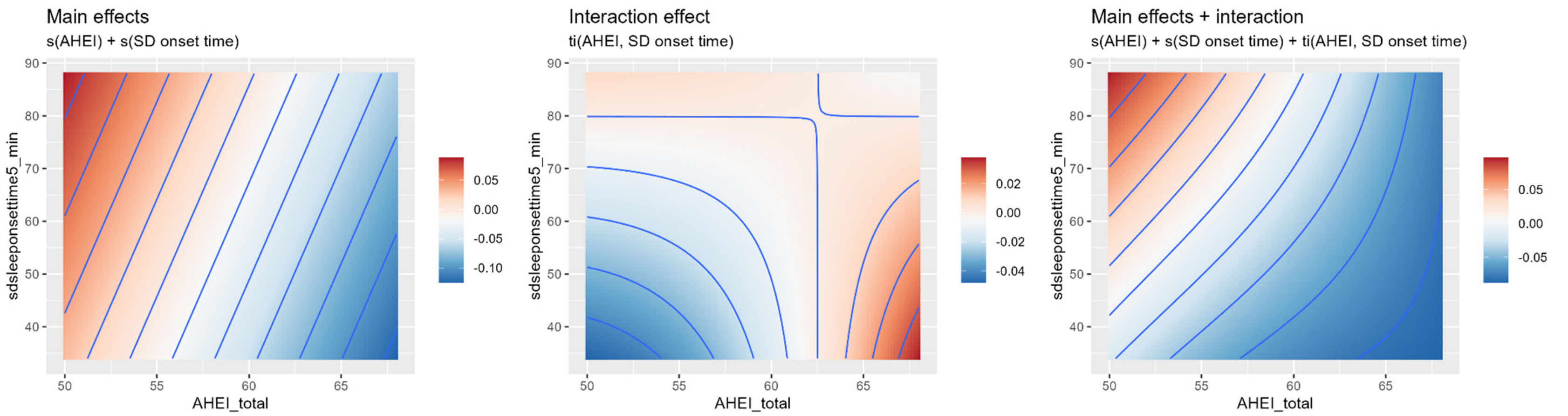

Figure S12. Interaction plot for diet quality and sleep timing regularity. The plotted range is restricted to show the 25th to 75th percentiles for both variables. The plot on the left shows the smoothed main effects, the center plot shows the smoothed interaction (without the main effects), and the plot on the right gives the smoothed main effects plus the interaction. The color gradient reflects CVD risk based on the log(HR), where blue indicates lower risk and red indicates higher risk. Interpretation: the highest risk is observed for those with irregular sleep (high SD sleep onset time) and low diet quality (low AHEI). The lowest risk is observed for those with the most regular sleep (lowest SD sleep onset time) and highest diet quality (high AHEI) when we consider only the main effects, but this shifts slightly towards a medium–high diet quality when the interaction is included. The fact that the left and right plots are similar reinforces the fact that the interaction does not have a big effect and that it is not statistically significant.

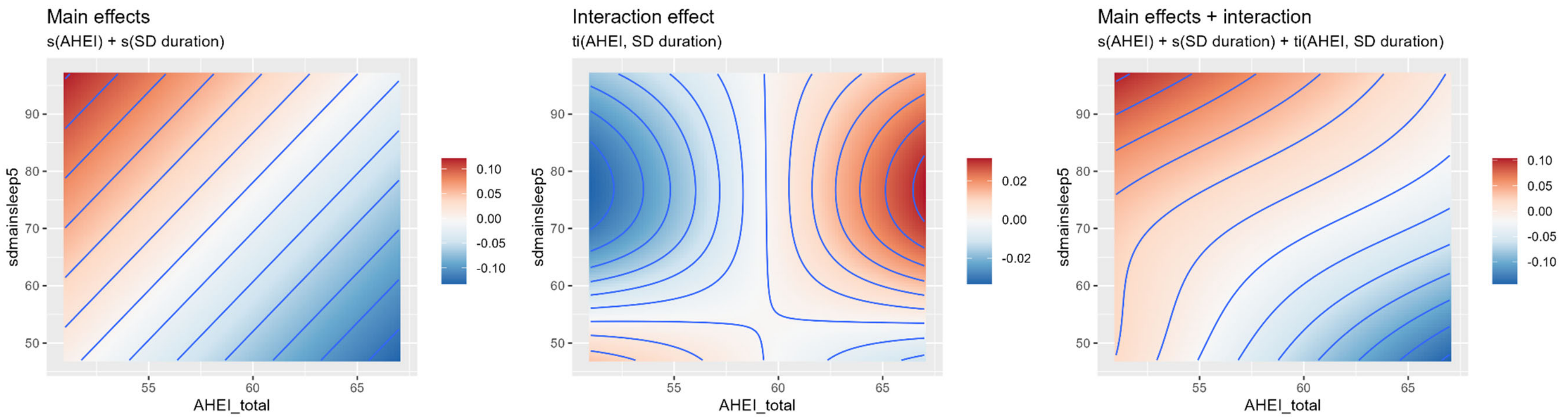

Figure S13. Interaction plot for diet quality and sleep duration regularity. The plotted range is restricted to show the 25th to 75th percentiles for both variables. The plot on the left shows the smoothed main effects, the center plot shows the smoothed interaction (without the main effects), and the plot on the right gives the smoothed main effects plus the interaction. The color gradient reflects CVD risk based on the log(HR), where blue indicates lower risk and red indicates higher risk. Interpretation: the highest risk is observed for those with irregular sleep (high SD sleep duration) and low diet quality (low AHEI). The lowest risk is observed for those with the most regular sleep (lowest SD sleep duration) and highest diet quality (high AHEI). The fact that the left and right plots are very similar reinforces the fact that the interaction does not have a big effect and that it is not statistically significant.

References

1. Chiuve, S. E. *et al.* Alternative Dietary Indices Both Strongly Predict Risk of Chronic Disease. *The Journal of Nutrition* **142**, 1009–1018 (2012).
2. VanderWeele, T. J. & Knol, M. J. A Tutorial on Interaction. *Epidemiologic Methods* **3**, 33–72 (2014).
